# Autism-like phenotypes and increased NMDAR2D expression in mice with KDM5B histone lysine demethylase deficiency

**DOI:** 10.1126/sciadv.adq6577

**Published:** 2026-05-20

**Authors:** Leticia Pérez-Sisqués, Shail U. Bhatt, Angela Caruso, Josephine L. Robb, Alex P. A. Donovan, Rosemary Bamford, Alejo Torres-Cano, Shoshana Spring, Eleanor Hendy, Talia E. Gileadi, Martyna Panasiuk, Jed Trengove, Neeru Jindal, Mohi U. Ahmed, Mara Sabbioni, Joyce Taylor-Papadimitriou, Diana Cash, Nicholas Clifton, Jacob Ellegood, Laura C. Andreae, Jason P. Lerch, Maria Luisa Scattoni, K. Peter Giese, Cathy Fernandes, M. Albert Basson

**Affiliations:** ^1^ Centre for Craniofacial and Regenerative Biology, Guy’s Hospital, King’s College London, London SE1 9RT, UK.; ^2^ MRC Centre for Neurodevelopmental Disorders, New Hunt’s House, King’s College London, London SE1 1UL, UK.; ^3^ Research Coordination and Support Service, Istituto Superiore di Sanità, 00161 Rome, Italy.; ^4^ Clinical and Biomedical Sciences, University of Exeter Medical School, Hatherly Laboratories, Prince of Wales Road, Exeter EX4 4PS, UK.; ^5^ MRC Laboratory of Molecular Biology (LMB), Cambridge Biomedical Campus, Francis Crick Avenue, Trumpington, Cambridge CB2 0QH, UK.; ^6^ Centre for Gene Therapy and Regenerative Medicine, King’s College London, London SE1 9RT, UK.; ^7^ Mouse Imaging Centre (MICe), Hospital for Sick Children, Toronto, Ontario M5T 3H7, Canada.; ^8^ Comprehensive Cancer Centre, King’s College London, Great Maze Pond, London SE1 1UL, UK.; ^9^ Biomarker Research And Imaging in Neuroscience (BRAIN) Centre, Department of Neuroimaging, King’s College London, London, SE5 9NU, UK.; ^10^ Bloorview Research Institute, Holland Bloorview Kids Rehabilitation Hospital, Toronto, Ontario M4G 1R8, Canada.; ^11^ Wellcome Centre for Integrative Neuroimaging, Nuffield Department of Clinical Neurosciences, University of Oxford, Oxford OX3 9DU, UK.; ^12^ Department of Basic and Clinical Neuroscience, Maurice Wohl Clinical Neuroscience Institute, King’s College London, 5 Cutcombe Rd, London SE5 9RT, UK.; ^13^ Social, Genetic and Developmental Psychiatry Centre, Institute of Psychiatry, Psychology and Neuroscience, King’s College London, 16 De Crespigny Park, London SE5 8AB, UK.

## Abstract

Loss-of-function mutations in genes encoding lysine demethylases specific for trimethylated lysine 4 of histone 3 (H3K4me3) are associated with neurodevelopmental conditions, including autism spectrum disorder (ASD) and intellectual disability (ID). To study the role of KDM5B (lysine demethylase 5B)–mediated H3K4me3 demethylation, we investigated neurodevelopmental phenotypes in mice without KDM5B demethylase activity. These mice exhibited autism-like behaviors and increased brain size. H3K4me3 levels and the expression of neurodevelopmental genes were increased in the developing *Kdm5b* mutant neocortex. Increased H3K4me3 levels at the promoter and associated expression of the *Grin2d* gene were associated with increased levels of *N*-methyl-d-aspartate receptor subunit 2D (NMDAR2D) protein in synaptosomes isolated from the early postnatal *Kdm5b*-deficient neocortex. Treating mice with the NMDAR antagonist memantine rescued deficits in ultrasonic vocalizations. These findings suggest that increased H3K4me3 levels and associated *Grin2d* gene up-regulation disrupt brain development and function, leading to socio-communication deficits and identify a potential therapeutic target for neurodevelopmental disorders associated with KDM5B deficiency.

## Teaser

Inhibitors targeting NMDA receptors may represent viable therapies for KDM5B neurodevelopmental disorders.

## INTRODUCTION

Mutations and variants in >50 genes encoding proteins associated with chromatin are linked to neurodevelopmental disorders (*1*). Several of these factors function as regulators of chromatin modified by histone 3 lysine 4 trimethylation (H3K4me3), a posttranslational modification typically found at active gene promoters (*2*, *3*). As H3K4me3 is permissive for transcription and linked to transcriptional elongation and output (*4*), mutations in genes encoding histone methyltransferases that catalyze H3K4 methylation, or in genes encoding “reader” proteins that mediate downstream effects of H3K4me3, are predicted to be associated with reduced transcription (*5*, *6*). Examples include chromodomain helicase DNA-binding factor 8 (CHD8), an adenosine 5′-triphosphate–dependent chromatin remodeler recruited to H3K4me2/3, and lysine methyltransferase 2A (KMT2A, an H3K4 methyltransferase. Mutations of the *CHD8* or *KMT2A* genes cause defined syndromes characterized by autism spectrum disorder (ASD) with high penetrance (*7*–*14*). H3K4me2 levels were found to be reduced in a small set of brain samples from idiopathic ASD individuals and genetically defined ASD mouse models (*15*). Restoring normal H3K4me2 levels in these mouse models by treatment with H3K4-specific lysine demethylases rescued ASD-associated phenotypes, suggesting that the reduced H3K4me2 levels are responsible for these phenotypes (*15*).

Mutations in H3K4me3-specific demethylases of the lysine demethylase 5 (KDM5) family are also associated with ASD (*12*, *13*, *16*), suggesting that increased H3K4me3 and transcriptional output could also lead to ASD. A recent forward genetic study identified recessive *Kdm5a* mutations in mice with alterations in ultrasonic vocalization (USV) emissions and nest building phenotypes and further reported evidence for social, repetitive, and cognitive phenotypes in *Kdm5a^−/−^* mice (*17*). Hayek *et al.* further showed that individuals with recessive and heterozygous *KDM5A* mutations exhibited ASD, developmental delay, and intellectual disability (ID). Mutations in the X-linked KDM5C gene that either disrupt its demethylase activity, or keep enzymatic activity intact, cause the intellectual disability Claes-Jensen syndrome in males (*18*, *19*). Recessive mutations in *KDM5B* cause a human syndrome associated with developmental delay, ASD, ID, hypotonia, craniofacial, and limb abnormalities (*20*). In contrast to loss-of-function variants in genes such as *CHD8* that are associated with ASD at high penetrance, heterozygous *KDM5B* mutations associated with ASD and developmental delay are not fully penetrant and are often inherited from apparently unaffected parents (*13*, *16*, *21*–*24*). Many ASD-associated missense mutations in *KDM5B* map to the JumonjiN (JmjN), A-T rich interaction domain (ARID) and JumonjiC (JmjC) domains that form the catalytic domain of the protein, suggesting that changes in demethylase activity are associated with the neurodevelopmental phenotypes in these individuals (*16*). Two recent studies have found a significant association between *KDM5B* protein-truncating variants and attention deficit hyperactivity disorder (ADHD) (*25*, *26*), suggesting that *KDM5B* loss-of-function variants predispose to a number of neurodevelopmental disorders.

KDM5B is essential for normal development with homozygous loss-of-function mutations in mice resulting in embryonic or significant early postnatal lethality (*23*, *27*, *28*). Similar to *Drosophila Kdm5*, the lysine demethylase activity of KDM5B is not essential for embryonic development, as a homozygous mouse model that lacks this activity is viable and fertile with only a subtle mammary gland phenotype reported to date (*28*, *29*). These findings are consistent with the observation that H3K4me3 levels only become dysregulated in *Kdm5b*-deficient brain tissue after embryonic day 17.5 (E17.5) (*27*). The reason why *Kdm5b* deficiency has little effect on H3K4me3 levels in the brain in the midgestation embryo is not known, but the most likely possibility is compensation by other KDM5 family members.

In addition to mutations in genes encoding chromatin remodeling factors and synaptic proteins, de novo variants in genes encoding N-methyl-D-aspartate receptor (NMDAR) subunits have been identified in ASD probands, and both reduced and elevated NMDAR activity have been implicated in ASD (*30*, *31*). NMDARs are ligand-gated cation channels that mediate excitatory synaptic transmission (*32*). Increased NMDAR function during the early postnatal period [before postnatal day 21 (PND21)] has been reported in specific ASD rodent models, including a *Shank2^e6–7^* mutant and valproic acid (VPA) exposure model (*33*, *34*). The expression of the genes encoding NMDAR subunits is developmentally regulated. For instance, *Grin2d*, encoding the NMDAR2D subunit, is not expressed in the prenatal neocortex but is up-regulated early postnatally, with peak expression at PND7, whereafter expression declines (*35*). Treatment of *Shank2^e6–7^* mutant mice at the time of NMDAR overactivity (PND7 to PND21) with a noncompetitive NMDAR antagonist, memantine (*36*), rescued the sociability phenotype (*34*). Memantine treatment of adult *IRSp53* knockout mice was also able to restore social interactions but not a hyperactivity or adult USV phenotype (*37*). The NMDAR antagonist agmatine rescued the social, hyperactivity, and repetitive behavioral phenotypes in a VPA-exposed rat ASD model (*38*). Reduced NMDAR function has been linked to social deficits in *Shank3* mutant mice, and treatment with histone deacetylase inhibitors rescued these deficits and restored NMDAR expression and function (*39*, *40*).

To test whether dysregulation of the H3K4me3 demethylase activity of KDM5B is sufficient to cause ASD, we characterized mice that lack KDM5B demethylase activity (*29*). These mice were found to exhibit ASD-like behavioral phenotypes. Molecular studies revealed increased H3K4me3 levels and gene expression abnormalities in the early postnatal brain. These changes included an up-regulation of the NMDAR2D subunit, encoded by the *Grin2d* gene, in specific cell types. Treatment with memantine increased USV emission in pups and reduced excessive digging behavior in adult mice, suggesting that NMDAR hyperactivation may be responsible for ASD-like behavioral phenotypes.

## RESULTS

### KDM5B is an H3K4me3 demethylase in the developing neocortex

**Fig. 1.**
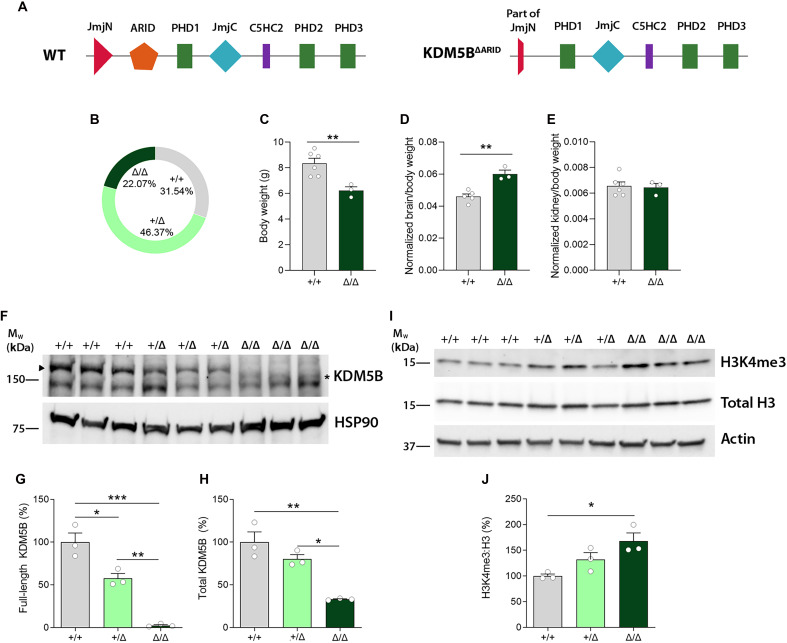
Developmental alterations and KDM5B expression in control and ΔARID mice. (**A**) Schematic representations of mouse KDM5B wild-type (WT) and ΔARID protein domains. Transgenic *Kdm5b* Δ*ARID* mice carry a deletion of the entire ARID domain and a truncation of the carboxyl end of the JmjN domain. (**B**) Genotyping frequencies show no statistically significant differences between genotypes (chi-square test) at PND21. (**C** to **E**) *Kdm5b* Δ*ARID* homozygous mice (*Kdm5b*^Δ/Δ^) exhibit decreased weight and increased brain-to-body weight ratios with no difference in kidney-to-body weight ratios. *N* = 5 to 6 +/+ and 3 Δ/Δ mice. (**F** to **H**) Estimation of full-length (G) and total (H); full-length and truncated) KDM5B protein expression by Western blot in neocortical samples extracted from PND5 animals. Heat shock protein 90 (HSP90) was used as loading control. Arrowhead, full-length KDM5B isoform; asterisk, truncated ΔARID protein. *M*_w_, molecular weight. (**I** and **J**) H3K4me3 and total H3 levels in WT, heterozygous, and ΔARID homozygous mice in neocortical samples extracted from PND5 animals. WT (+/+) and ΔARID heterozygous (+/Δ) and homozygous (Δ/Δ) mice. Data are shown as percentage versus WT average. *N* = 3 +/+ and 3 Δ/Δ mice. Data are shown as mean ± SEM and are analyzed with one-way analysis of variance (ANOVA), followed by Tukey’s multiple-comparison test. *P < 0.05, **P < 0.01, and ***P < 0.001.

To study the function of the histone demethylase KDM5B and avoid complications due to embryonic or early postnatal lethality, we established a mouse line in which exons 2 to 4 were deleted, resulting in the truncation of the carboxyl end of the JmjN domain and deletion of the entire ARID domain (Fig. 1A). This mutation specifically disrupts the H3K4me3 demethylase activity of the protein, leaving the developmental functions of KDM5B required for embryonic development and postnatal survival intact (*28*, *29*). Albert *et al.* (*27*) have shown that *Kdm5b* deletion only affects H3K4me3 levels from late embryonic brain development, so we focused our analysis on postnatal stages.

Homozygous *Kdm5b*^Δ*ARID/*Δ*ARID*^ (*Kdm5b*^Δ*/*Δ^) mice on a C57BL/6J background were born within expected ratios from *KDM5B*^*+/*Δ^ intercrosses (+/+: 31.54%, +/Δ: 46.37%, Δ/Δ: 22.07%; *n* = 69, χ^2^ test, *P* = 0.6077) with no reduction in postnatal survival (Fig. 1B). When examined at PND12, homozygous mutant mice displayed decreased body weight compared to wild-type (WT) littermates, suggesting some effect on their ability to grow or thrive (Fig. 1C). At this stage, mutant mice exhibited increased brain-to-body weight ratios (Fig. 1D), while the relative size of other vital organs was not affected (Fig. 1E), suggesting specific neurodevelopmental functions for KDM5B.

To determine the effects of the ΔARID mutation on KDM5B protein in the brain, we visualized KDM5B by immunoblot in brain lysates. The shorter KDM5B ΔARID protein was detected in hippocampal and neocortical samples from PND5 control, *Kdm5b*^*+/*Δ^, and *Kdm5b*^Δ*/*Δ^ pups, with the expected reduced molecular weight (Fig. 1F). In line with previous results (*29*), full-length KDM5B protein was reduced by 50% in the neocortex of *Kdm5b*^*+/*Δ^ mice and absent in *Kdm5b*^Δ*/*Δ^ mice (Fig. 1, F and G). Total (full-length plus ΔARID) KDM5B protein levels were reduced in the neocortex of homozygous mice compared to WT littermates (Fig. 1, F and H). Thus, in addition to the reported lack of demethylase activity, our data suggest that the ΔARID protein is also slightly less stable than the full-length protein, although the possibility that the antibody is less efficient at detecting the mutant protein cannot be excluded.

The ΔARID protein lacks demethylase activity in in vitro assays (*29*). To determine whether KDM5B functions as an H3K4me3 demethylase in the developing brain, and whether steady-state H3K4me3 levels are altered in *KDM5B*^Δ*/*Δ^ mice, we compared H3K4me3 levels in brain tissue from WT, heterozygous, and homozygous mice. We detected a significant increase in H3K4me3 levels in neocortical samples from homozygous, but not heterozygous mice (Fig. 1, I and J).

### Autism-like behavioral phenotypes in *Kdm5b*^Δ*/*Δ^ mice

**Fig. 2.**
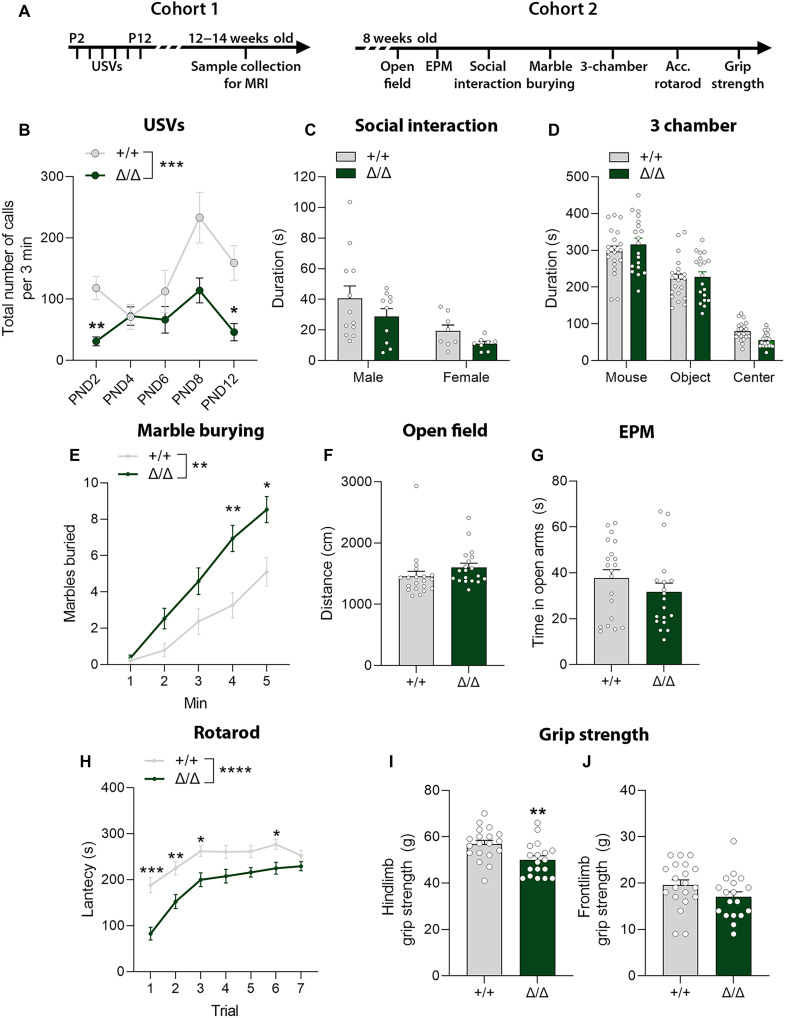
Behavioral assessment of *Kdm5b*^Δ/Δ^ mice. Behavioral assessment of a cohort of neonatal (B) (+/+ *N* = 3 males and 7 females; Δ/Δ *N* = 6 males and 6 females) and adult mice [(C) to (J)] (+/+ *n* = 11 to 12 males and 7 to 8 females; Δ/Δ *n* = 10 to 11 males and 6 to 8 females). (**A**) Experimental time course. Different cohorts were used for experiments with neonatal and adult mice. (**B**) Total number of USVs during 3 min of testing sessions on indicated PNDs. Repeated-measures two-way ANOVA interaction: *F*_4,80_ = 2.866, *P = 0.0284; genotype effect: *F*_1,20_ = 15.03, ***P = 0.0009. (**C**) Duration, in seconds, of social investigation, defined as total duration of sniffing above the shoulders of a conspecific mouse. Two-way ANOVA sex effect: *F*_1,34_ = 9.785, **P = 0.0036. (**D**) Time spent, in seconds, in each chamber in the three-chamber sociability test. (**E**) Number of marbles buried during a 5-min test. Repeated-measures two-way ANOVA interaction: *F*_4,136_ = 5.894, ****P = 0.0002; genotype effect: *F*_1,34_ = 11.08, **P = 0.0021. (**F**) Distance traveled in the outer area of an open field arena during a 5-min test. (**G**) Time spent in the open arms of the EPM is shown. (**H**) Mean latency of mice to fall from the accelerating rotarod during seven trials in 1 day. Repeated-measures two-way ANOVA interaction: *F*_6,222_ = 2.944, **P = 0.0088; genotype effect: *F*_1,37_ = 21.54, ****P < 0.0001. (**I** and **J**) Hind- and frontlimb grip strength measurements. Data are shown as mean ± SEM and were analyzed with two-way ANOVA [(C) and (D)], repeated-measures ANOVA [(B), (E), and (H)] followed by Šidák’s post hoc test, and Student’s *t* test [(F), (G), (I), and (J)]. *P < 0.05, **P < 0.01, and ***P < 0.001. Acc. rotarod, accelerating rotarod.

To determine whether a reduction in KDM5B levels and lack of KDM5B demethylase activity are sufficient to cause behavioral phenotypes in mice, we assessed sociocommunicative, repetitive, anxiety, and motor behaviors (Fig. 2A). Both male and female animals were tested (fig. S1), and data were combined when no significant sex differences were observed (Fig. 2). Homozygous pups emitted significantly fewer USVs upon separation from the mother (Fig. 2B). Homozygous adults exhibited no significant differences in social approach and social investigation compared to WT littermates in the social investigation and three-chamber tests, respectively (Fig. 2, C and D). In the marble burying test, a measure of repetitive behaviors, mutant mice buried marbles significantly faster than WT littermates, indicative of compulsive, repetitive digging behaviors (Fig. 2E). General locomotion and anxiety levels did not differ between genotypes in an open field arena (Fig. 2F and fig. S1, E and F). As further confirmation, no differences were found in the elevated plus maze (EPM) test (Fig. 2G), another test for anxiety. Homozygous mice showed reduced performance compared to WT animals in the accelerating rotarod task (Fig. 2H). *Kdm5b*^Δ*/*Δ^ mice exhibited reduced hindlimb grip strength (Fig. 2, I and J), which may have contributed to their poor performance on the rotarod test and is consistent with a report of hypotonia in an individual with recessive *KDM5B* mutations (*41*).

### Structural brain anomalies associated with KDM5B deficiency

**Fig. 3.**
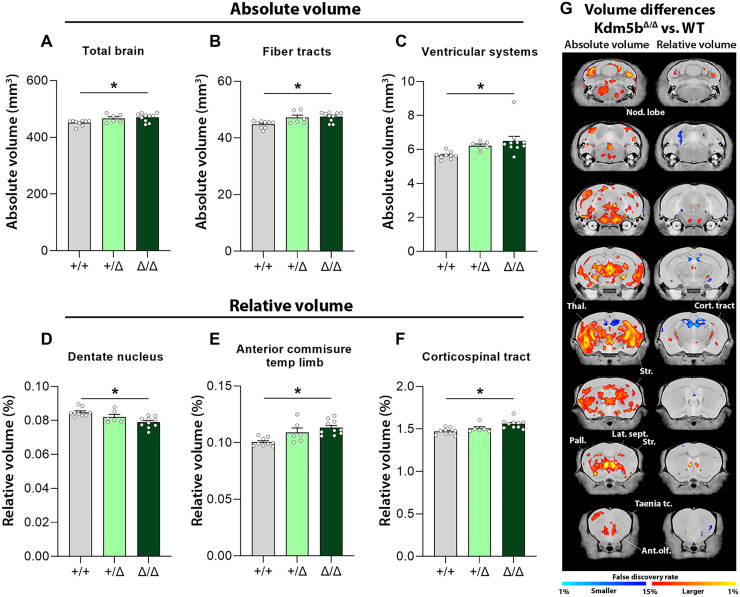
*Kdm5b*^Δ/Δ^ mutant mice display megalencephaly and altered regional volume of different brain areas. (**A** to **C**) MRI analysis revealed significant increases in absolute brain size together with increased regional volume of different brain areas such as the fiber tracts and the ventricular systems. (**D** to **F**) Correction for absolute brain volume revealed significant changes in volume in the dentate nucleus, corticospinal tract, and anterior commissure of the temporal limb. (**G**) Voxel-wise differences in volume (absolute, left; relative, right) between WT and *Kdm5b*^Δ/Δ^ mutant littermates. Data are shown as mean ± SEM. Multiple comparisons (all brain regions) were controlled for using the false discovery rate (FDR) (see tables S1 to S4). *FDR < 0.05. *N* = 9 +/+, 5 +/Δ, and 10 Δ/Δ animals. Nod. lobe, cerebellar nodular lobe; Thal., thalamus; Cort. tract, corticospinal tract; Str., striatum; Pall., pallidum; Lat. Sep., lateral septum; Taenia tc., taenia tecta; Ant. olf., anterior olfactory nucleus.

Intact brains were collected from the mice that underwent USV phenotyping (Fig. 2A). Structural magnetic resonance imaging (MRI) was performed to identify changes in absolute and relative volumes. We assessed 290 brain regions with divisions across the cortex, subcortical areas, cerebellum, brain stem, ventricular systems, and fiber tracts (see tables S1 to S4 for full results). A 4.2% (±1.06%) increase in brain volume was detected in homozygous mutant animals (Fig. 3A). Moreover, absolute volumes for the fiber tracts and the ventricular systems were also increased in *Kdm5b*^Δ*/*Δ^ mice (6.02 ± 0.62 and 15.23 ± 10.47, respectively) (Fig. 3, B and C). Alterations within the fiber tracts included the anterior commissure (temporal limb) and the corticospinal tract. Mutant mice showed increases in volume across cortical areas such as the anterior olfactory nucleus, the endopiriform nucleus, the taenia tecta, the infralimbic area, the primary somatosensory area associated to the nose, and the dorsal auditory area (11.28 ± 1.9, 8.57 ± 2.04, 8.84 ± 1.56, 11.17 ± 0.36, 7.39 ± 3.06, and 9.34 ± 0.61% increases, respectively) (Fig. 3G and tables S1 and S3). *Kdm5b*^Δ*/*Δ^ mice also showed robust increases in volume in cerebral nuclei such as the striatum (+7.52 ± 1.52%), dorsal pallidum (+9.04 ± 3.38%), lateral septum (+7.32 ± 0.43%), the thalamus (+5.54 ± 1.77%), and in the cerebellar nodular lobe (+15.67 ± 2.07%) (Fig. 3G and tables S1 and S3).

After correction for total brain volume, relative volumes were significantly reduced in the dentate nucleus (−6.84 ± 0.9%) but still significantly larger in the anterior commissure of the temporal limb (+12.64 ± 2.18%) and in the corticospinal tract (+6.16 ± 0.68%) (Fig. 3, D to F). Voxel-wise differences showed similar trends (Fig. 3G). We did not detect significant differences between WT and heterozygous animals in either absolute or relative volumes, although similar trends were apparent compared to homozygotes (fig. S2 and tables S2 and S4). Accordingly, brain volume negatively correlated with WT *Kdm5b* copy number (fig. S2, B to G).

### KDM5B regulates the expression of neurodevelopmental genes

**Fig. 4.**
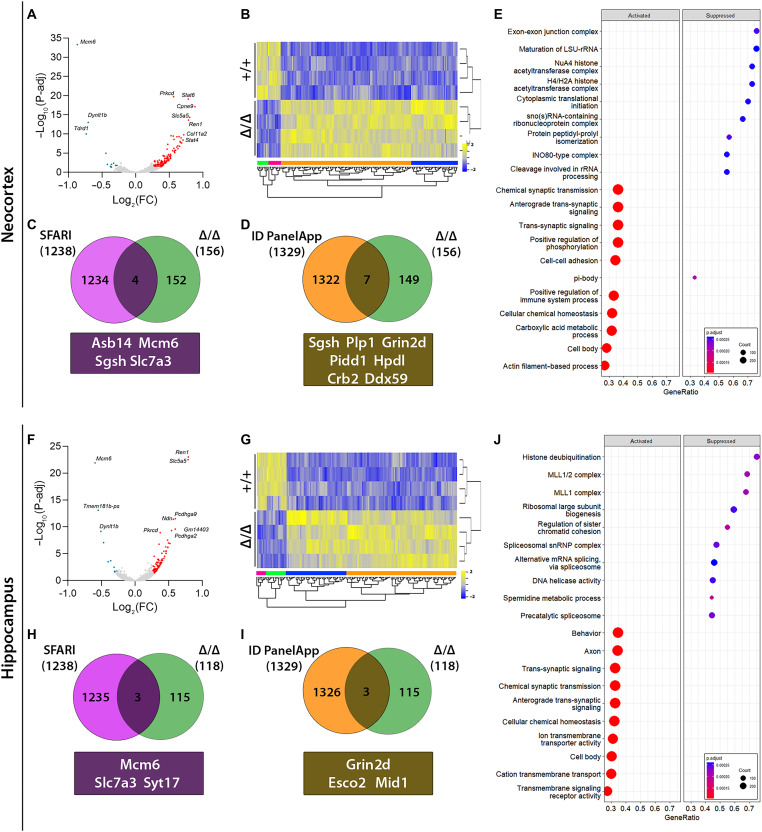
Gene expression changes in PND5 *Kdm5b*^Δ/Δ^ neocortex and hippocampus determined by RNA-seq. (**A** and **F**) Volcano plot displaying gene expression changes detected by DESeq2 in neocortex (A) and hippocampus (F). Each point represents an individual gene, and all DEGs (FDR < 0.05) are highlighted in red (up-regulated) or blue (down-regulated). The top 10 DEGs are labeled next to their corresponding position. *N* = 4 mice per genotype, 2 males and 2 females. FC, fold change. (**B** and **G**) Heatmap of sequenced genes in neocortical (B) and hippocampal (G) samples. Yellow: up-regulated; blue: down-regulated. (**C** and **H**) Venn diagrams showing the extent of overlap between DEGs (FDR < 0.05), and ASD-associated genes obtained from the SFARI Gene database. The overlapping genes are shown below the diagram. (**D** and **I**) Venn diagrams showing the extent of overlap between DEGs (FDR < 0.05), and ID-associated genes obtained from the Genomics England PanelApp intellectual disability database. The overlapping genes are shown below the diagram. (**E** and **J**) Gene set enrichment analyses in the neocortex (E) and hippocampus (J) with the top 10 most significant Gene Ontology (GO) terms for both up-regulated (activated) and down-regulated (suppressed) genes.

The increased H3K4me3 observed in the PND5 neocortex of *Kdm5b*^Δ/Δ^ mice (Fig. 1G) is predicted to result in increased gene expression. To test this hypothesis, we compared gene expression in *Kdm5b*^Δ/Δ^ and WT PND5 neocortical and hippocampal tissues by RNA sequencing (RNA-seq) (tables S5 and S6). This analysis identified 156 differentially expressed genes [DEGs; log fold change > 0.26; false discovery rate (FDR) < 0.05] in the neocortex (Fig. 4A) and 118 DEGs in the hippocampus (Fig. 4F). The transcriptomic differences between the two genotypes were visualized in volcano plots and heatmaps, where the majority of transcripts were up-regulated in *Kdm5b*^Δ*/*Δ^ mice, both in the neocortex (Fig. 4, A and B) (88%) and hippocampus (Fig. 4, F and G) (85%), in support of our hypothesis. Fourty-four of the DEGs were shared between the neocortex and the hippocampus (fig. S4 and tables S9 and S10).

Gene set enrichment analyses were performed to identify potential biological functions affected in *Kdm5b*^Δ/Δ^ mutants. This analysis identified a significant increase in synaptic signaling processes, in both neocortical and hippocampal datasets, together with a significant overall decrease in genes related to histone complexes and RNA processing (Fig. 4, E and J). A few DEGs were known SFARI ASD-associated genes (Fig. 4, C and H) and ID PanelApp intellectual disability–associated genes (Fig. 4, D and I) (tables S7 and S8). These ASD/ID-associated genes included the most significantly down-regulated gene *Mcm6* (*12*, *13*, *42*), the down-regulated gene *Slc7a3*, which encodes the cationic amino acid transporter CAT3 (*43*), and the NMDAR subunit gene *Grin2d*, which was up-regulated. We validated the up-regulation of *Grin2d* and down-regulation of *Mcm6* by quantitative polymerase chain reaction (qPCR), noting that the apparent increase in *Grin2d* levels in the neocortex was not significant in this assay (fig. S3).

### Genome-wide changes in H3K4me3

**Fig. 5.**
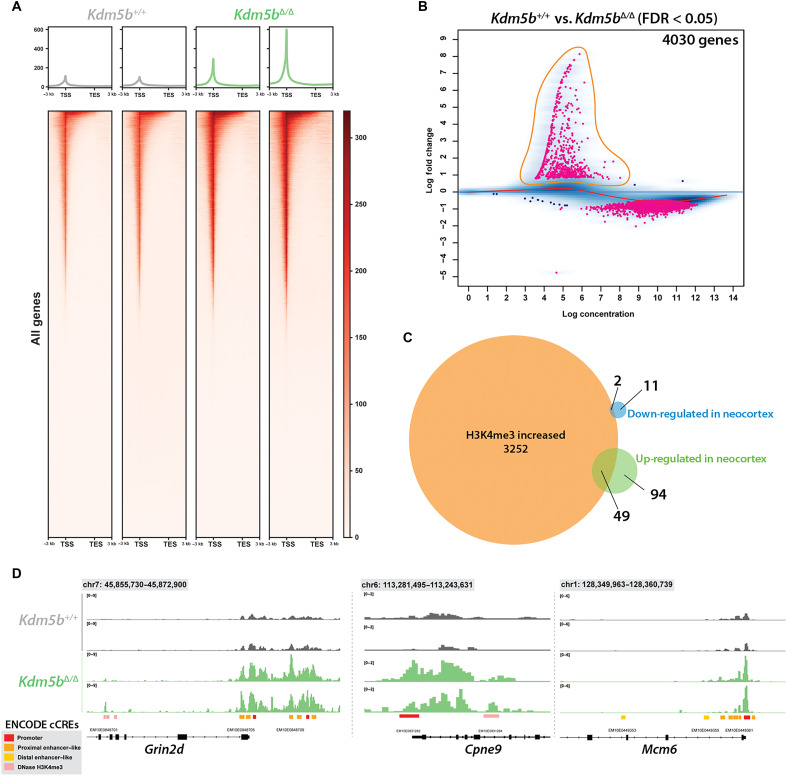
Quantitative CUT&Tag-seq mapping reveals genome-wide increases in H3K4me3 in the neocortex of *Kdm5b*^Δ/Δ^ mice. (**A**) Heatmaps of H3K4me3 enrichment around the TSS of all genes in *Kdm5b*^Δ/Δ^ and *Kdm5b*^+/+^ mice (*n* = 2 of each). (**B**) DiffBind MA plot identifying gene TSS with significantly (FDR < 0.05) altered enrichment between *Kdm5b*^Δ/Δ^ and *Kdm5b*^+/+^ mice. (**C**) Venn diagram overlap of genes identified as differentially expressed between *Kdm5b*^Δ/Δ^ and *Kdm5b*^+/+^ mice by bulk RNA-seq and those associated with significantly increased enrichment of H3K4me3 proximal to the TSS. (**D**) Integrative Genomics Viewer (IGV) genome browser visualization of H3K4me3 enrichment at the promoters of *Grin2d* and *Mcm6* showing increases in *Kdm5b*^Δ/Δ^ mice. ENCODE candidate cis-regulatory elements (cCREs) are shown to demarcate promoter elements. DNase, deoxyribonuclease; TES, transcription end site.

To identify genes with increased H3K4me3 levels at promoter regions and therefore most likely direct targets of KDM5B, we performed CUT&Tag sequencing (CUT&Tag-seq) from PND5 neocortices (*n* = 2 WT, *n* = 2 mutant). This genome-wide analysis showed a notable increase in H3K4me3 intensity at transcription start sites (TSSs) in the *Kdm5b*^Δ/Δ^ samples (Fig. 5A), consistent with our Western blot analysis (Fig. 1, I and J). Consistent with the function of KDM5B, the most pronounced changes in H3K4me3 involved increased H3K4me3, while genes with reduced H3K4me3 showed relatively small changes (Fig. 5B and table S10). An overlap with RNA-seq data found increased H3K4me3 at TSSs of 2 down-regulated genes, while 49 up-regulated genes showed increased H3K4me3, consistent with these being most likely directly regulated by KDM5B (table S11).

We visualized H3K4me3 changes at the promoters of three exemplary genes: up-regulated *Grin2d* and *Cpne9* and *Mcm6*, the most down-regulated gene in our bulk RNA-seq experiment (Fig. 5D). H3K4me3 levels were significantly increased at both promoters of the up-regulated genes (Fig. 5D and table S10). Thus, H3K4me3 changes correlated with gene expression changes for *Grin2d and Cpne9*, suggesting that both genes are most likely direct targets of KDM5B. H3K4me3 levels at the *Mcm6* promoter were not significantly changed in *Kdm5b* mutants (table S10), albeit that a trend for increased H3K4me3 levels was evident (Fig. 5D), suggesting that the down-regulated expression of *Mcm6* is mediated via a different mechanism than direct regulation of H3K4me3.

### Single nucleus RNA-seq reveals specific cell types contributing to *Grin2d* up-regulation in the *Kdm5b* mutant neocortex

**Fig. 6.**
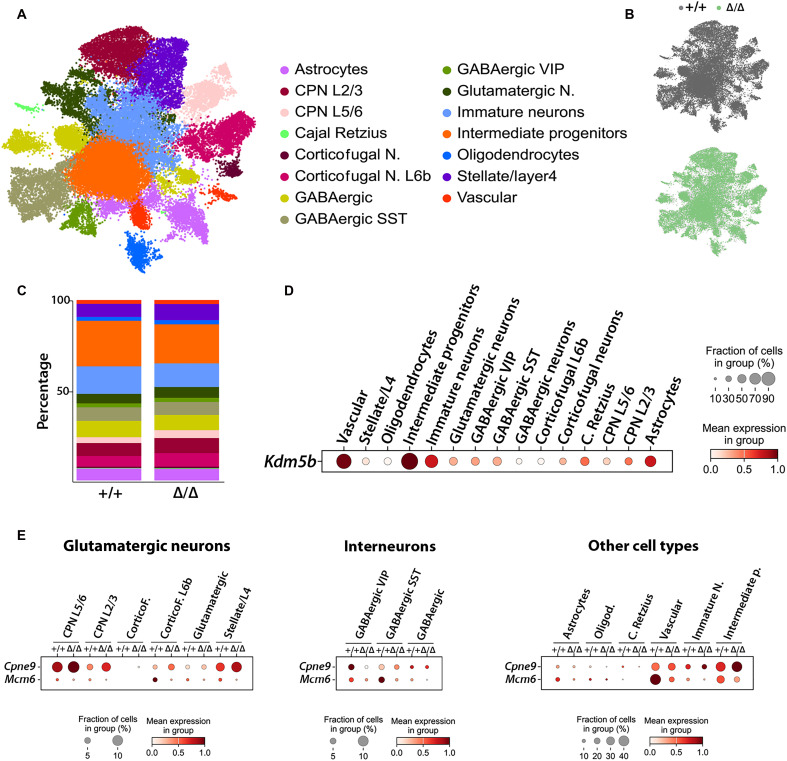
snRNA-seq of control and *Kdm5b* mutant neocortices at PND5. (**A**) Uniform manifold approximation and projection (UMAP) plot revealing the different cell identities in the neocortex. Data derive from eight +/+ and eight mutant mice. N, neuron. (**B**) Split UMAP revealing the contribution of the cells from each genotype to the broad cellular groups shown in (A). (**C**) Analysis of the cellular composition for each genotype does not reveal significant changes in the percentages of any cell population. (**D**) Dot plot showing the scaled expression of *Kdm5b* in the different cell populations. (**E**) Dot plots showing the scaled expression of *Cpne9* and *Mcm6* (top up- and down-regulated genes, respectively, in the bulk RNA-seq experiment) in the different cell populations, grouped as glutamatergic neurons, interneurons, and other cell types. CorticoF., corticofugal; C., Cajal; p., progenitors.

As pleiotropic chromatin regulators such as KDM5B are likely to perform different functions in multiple cell types in the developing brain, we performed single nucleus RNA-seq (snRNA-seq) on PND5 neocortices (*n* = 8 WT, *n* = 8 mutant) (Fig. 6A). After quality control filtering, data from 21,359 WT nuclei and 17,098 Δ/Δ nuclei were taken forward for analysis. Both genotypes included all major cell types (Fig. 6B), each characterized by the expression of their canonical markers (fig. S5A). We did not detect significant differences in the relative proportions of these cell types between control and mutant samples (Fig. 6C and fig. S5B). An analysis of *Kdm5b* expression revealed the highest expression in vascular cells, intermediate progenitors, immature neurons, and astrocytes (Fig. 6D). To validate our bulk RNA-seq data and identify the cell types responsible for gene expression differences, we analyzed cell type–specific expression of the top up- and down-regulated genes from the bulk RNA-seq experiment. *Cpne9* is up-regulated in glutamatergic neurons [callosal projection neurons (CPN), corticofugal neurons, and stellate/layer 4 cells], immature neurons, and intermediate progenitors. The small fraction (<5%) of GABAergic cells expression *Cpne6* showed a converse down-regulation in mutants (Fig. 6E). *Mcm6* was down-regulated in all cell types examined with the largest contribution from vascular cells and intermediate progenitors and, to a lesser extent, GABAergic interneurons (Fig. 6E).

The up-regulation of glutamate ionotropic receptor NMDA type subunit 2d (*Grin2d*), which encodes an NMDAR subunit (NMDAR2D), in *Kdm5b* mutants suggested a possible neurodevelopmental mechanism responsible for behavioral phenotypes. Microduplications at 19q13.33 that includes the *GRIN2D* gene (*44*) and both de novo gain- and loss-of-function mutations in *GRIN2D* are associated with neurodevelopmental disorders (*45*, *46*). Bidirectional changes in NMDAR signaling have been implicated in ASD (*30*). An analysis of publicly available RNA-seq data (*47*, *48*) confirmed that *Grin2d* expression is developmentally regulated, with expression peaking in late fetal development in humans and early postnatal development in mice (fig. S6).

**Fig. 7.**
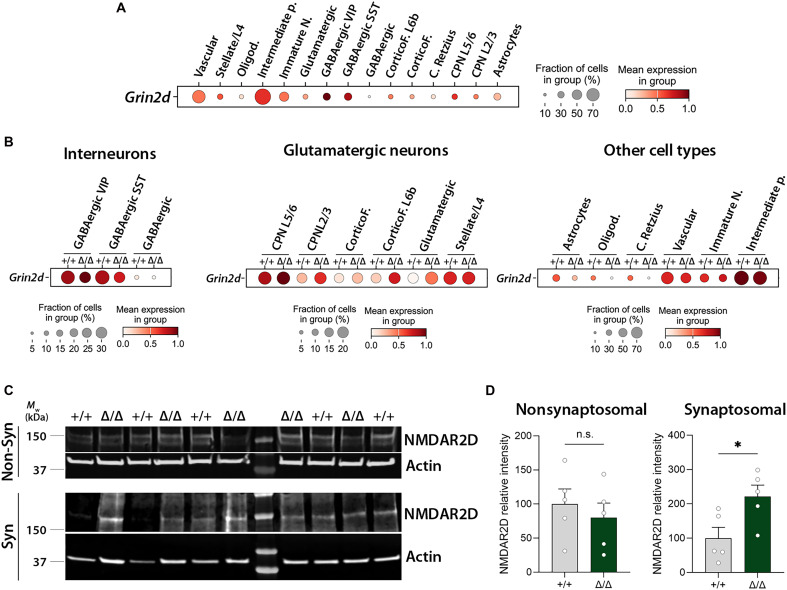
Characterization of the cell types contributing to increased *Grin2d* expression in *Kdm5b* mutant mice. (**A**) Dot plot showing the scaled expression levels of *Grin2d* in the different cell populations in our dataset. (**B**) Dot plots showing the scaled expression levels of *Grin2d* in the different cell types (grouped as interneurons, glutamatergic neurons, and other cell types) in control (+/+) and mutant (Δ/Δ) mice. (**C** and **D**) Neocortical samples from PND5 mice were dissected, and synaptosomes were isolated. Both the nonsynaptosomal and the synaptosomal fractions were subjected to Western blotting. Membranes were probed for NMDAR2D and β-actin. Molecular weights are shown on the left (C). The densitometric quantification of NMDAR2D levels in both fractions is shown in (D). *N* = 5 +/+ and 5 Δ/Δ mice. Syn, synaptosomal; Non-Syn, nonsynaptosomal; n.s., not significant, *P < 0.05.

An analysis of the snRNA-seq data showed that *Grin2d* was expressed in most vascular cells and intermediate progenitors (Fig. 7A), similar to *Kdm5b* (Fig. 6D). Expression levels were highest in vasoactive intestinal peptide-expressing (VIP+) interneurons, followed by somatostatin-expressing (SST+) interneurons, intermediate progenitors and CPN L5/6 neurons, albeit that a smaller fraction of interneurons appeared to express *Grin2d* (Fig. 7A). *Grin2d* expression was up-regulated in mutant mice in GABAergic VIP neurons and all glutamatergic cell types except for stellate/layer 4 neurons. Reduced *Grin2d* expression was observed in astrocytes, oligodendrocytes, Cajal Retzius cells, and intermediate progenitors in mutant samples. No evident differences were observed in vasculature or immature neurons (Fig. 7B).

Given the clear increase in *Grin2d* mRNA levels in glutamatergic cells, we next asked whether the levels of NMDAR2D protein, encoded by the *Grin2d* gene, were increased in synaptosomes isolated from the PND5 neocortex of *Kdm5b*^Δ/Δ^ mutants. NMDAR2D protein levels were quantified in purified synaptosomes and the remaining nonsynaptosomal fraction. NMDAR2D levels were not changed in the nonsynaptosomal fraction but were significantly increased in synaptosomes from mutant neocortices compared to WT tissue (Fig. 7, C and D). This result is in line with the clear changes in *Grin2d* expression in glutamatergic neurons in mutant mice, as synaptosomal preparations tend to be enriched for excitatory synapses (*49*). Together, these findings suggest that the increased expression of *Grin2d* results in up-regulated NMDAR2D that is preferentially incorporated into synaptic NMDARs.

### Treatment with an NMDAR antagonist can rescue behavioral phenotypes

Together, these observations suggested that increased NMDAR signaling during early postnatal development may underlie the behavioral phenotypes. To find direct evidence for elevated NMDAR signaling, we set out to measure AMPA:NMDA receptor ratios. However, we could not obtain reliable AMPA receptor (AMPAR) responses in PND5 mice, most likely due to the preponderance of “silent” synapses at this stage of development when most synapses only contain functional NMDAR, not AMPAR (*50*, *51*). We were able to measure AMPA:NMDA ratios at PND14 and found these to be the same in WT and mutant mice (fig. S7), consistent with the possibility that elevated NMDAR2D effects are transient.

**Fig. 8.**
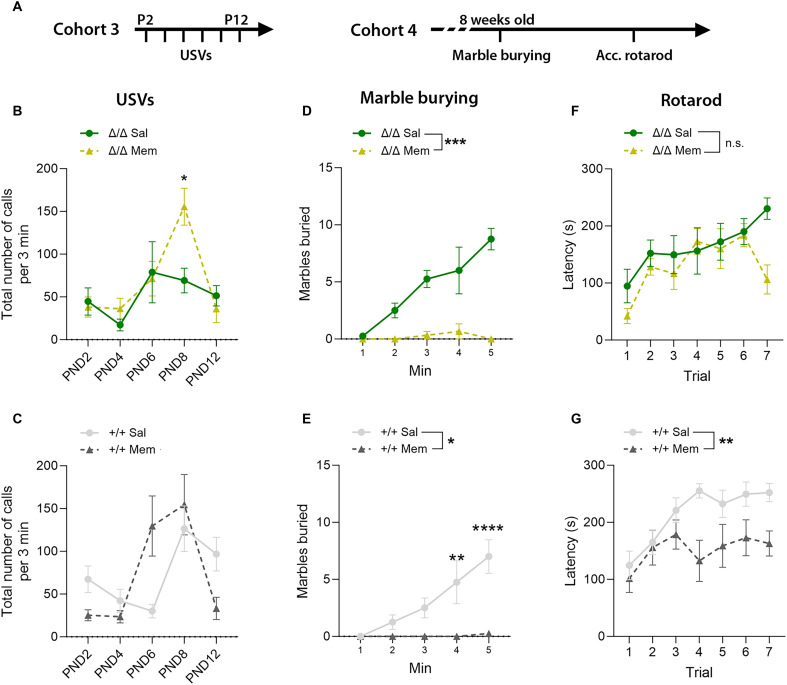
Memantine ameliorates neonatal communication deficits and digging behavior in *Kdm5b* mutant mice. (**A**) Experiment design. Two different cohorts were used for neonatal and adult tests. Memantine was administered 30 min before the start of the test each day. (**B**) Total number of USVs during 3-min testing sessions on indicated PNDs for *Kdm5b*^Δ/Δ^ mice. Repeated-measures two-way ANOVA interaction: *F*_2.6,31.24_ = 3.520, *P = 0.0314, followed by Šidák’s multiple-comparison test (PND8; *P = 0.0313). *N* = 6 Δ/Δ saline and 8 Δ/Δ memantine. (**C**) Total number of USVs during 3-min testing sessions on indicated PNDs for *Kdm5b*^+/+^ mice. Repeated-measures two-way ANOVA interaction: *F*_2.46,63.95_ = 6.208, **P = 0.0018. Šidák’s multiple-comparison test showed no significant differences at specific PNDs. *N* = 15 +/+ saline and 13 +/+ memantine. (**D** and **E**) Number of marbles buried during a 5-min test with 2-month-old mice. Repeated-measures two-way ANOVA treatment effect: *F*_1,5_ = 99.5, ***P = 0.0002 (D) and *F*_1,6_ = 13.56, *P = 0.0103 (E) and interaction effect: *F*_1.314,6.570_ = 5.12, **P = 0.0547 (D) and *F*_4,24_ = 8.387, ***P = 0.0002 (E), followed by Šidák’s multiple-comparison test. *N* = 4 +/+ saline, 4 +/+ memantine, 4 Δ/Δ saline, and 3 Δ/Δ memantine. (**F** and **G**) Mean latency of mice to fall from the rotarod during seven trials in 1 day. Repeated-measures two-way ANOVA treatment effect: *F*_1,11_ = 1.996, *P* = 0.1854 (F) and *F*_1,14_ = 10.48, **P = 0.006 (G) and interaction effect: *F*_4.166,45.83_ = 1.9, *P* = 0.1240 (F) and *F*_3.304,46.26_ = 1.524, *P* = 0.2178 (G). *N* = 10 +/+ saline, 6 +/+ memantine, 7 Δ/Δ saline, and 6 Δ/Δ memantine. Memantine doses were 5.6 mg/kg for neonatal animals in the USV test and 10 mg/kg for adult mice. Memantine or saline was administered 30 min before the start of the test. Data are shown as mean ± SEM. Sal, saline, Mem, memantine.

To find functional evidence that elevated NMDAR signaling in the early postnatal brain is responsible for behavioral phenotypes in *Kdm5b* mutants, we asked whether pharmacological inhibition of NMDARs could rescue these phenotypes. As NMDAR inhibition might be a potential therapeutic approach to explore in our *Kdm5b*-deficient mouse model, we decided to treat mice with the NMDAR antagonist memantine, which has an acceptable safety profile in humans, can cross the blood-brain barrier, and has shown some promise in the treatment of epileptic encephalopathy linked to increased NMDAR (*52*). As memantine has a half-life of <4 hours in rodents (*53*), memantine was administered 30 min before testing, in agreement with other studies (*37*, *54*–*57*). Memantine (5.6 mg/kg) administration by intraperitoneal injection 30 min before the test had a significant effect on USV production [repeated-measures three-way analysis of variance (ANOVA) interaction effect; *F*_2.567,97.54_ = 2.863, *P = 0.0487]. Considering genotypes separately, memantine significantly increased the number of ultrasonic calls emitted by *Kdm5b* mutants in response to maternal separation (repeated-measures two-way ANOVA interaction effect: *F*_2.6031,31.237_ = 3.520, *P = 0.0134), with a significant difference at PND8 (Fig. 8B), the peak of USV emission (see Fig. 2B). Memantine treatment of WT animals resulted in a significant shift of the developmental trajectory of USV production (repeated-measures two-way ANOVA interaction effect: *F*_2.46,63.95_ = 6.208, **P = 0.0018, with no significant effect on the number of USVs produced by WT animals on a particular day) (Fig. 8C).

Memantine treatment (10 mg/kg) of adult mice has been shown previously to strongly suppress repetitive digging behavior in the marble burying test in WT mice (*54*, *55*). We reproduced these findings by finding that marble burying was almost completely abrogated in both the *Kdm5b*^Δ/Δ^ mutant mice and WT littermates (repeated-measures three-way ANOVA treatment effect: *F*_1,11_ = 56.39, ***P < 0.0001) with no significant time × genotype × treatment interaction (Fig. 8, D and E). These observations suggest that NMDAR activity is a critical requirement for repetitive digging behaviors in mice, regardless of genotype. We also found that memantine treatment had a significant effect on motor performance in the accelerating rotarod test (repeated-measures three-way ANOVA treatment effect: *F*_1,25_ = 10.09, **P = 0.0039), with no significant time × genotype × treatment interaction effect. Considering the genotypes separately, memantine reduced the motor performance of WT animals in the accelerating rotarod test (Fig. 8G) but had no significant effect on the performance of mutant mice (Fig. 8F). These findings suggest that NMDAR antagonist treatment can rescue sociocommunicative deficits in *Kdm5b* mutants in the early postnatal period when *Grin2d* expression peaks and expression is elevated. Although memantine treatment in adults can strongly suppress repetitive behaviors, these effects are not specific to *KDM5B* deficiency. Together, these observations suggest that an NMDAR-mediated mechanism is responsible for the early postnatal behavioral phenotypes in *Kdm5b* mutants.

## DISCUSSION

In this study, we characterized the neurodevelopmental phenotypes of a mouse model homozygous for a hypomorphic, demethylase-deficient allele of *Kdm5b* on a C57BL/6J genetic background. These mice exhibited sociocommunicative, repetitive, and motor phenotypes consistent with the association of KDM5B loss-of-function variants with ASD and motor stereotypies (*12*, *58*–*60*). Steady-state H3K4me3 levels were elevated across the genome, and a number of genes were differentially expressed in the early postnatal neocortex of these mice compared to WT littermates. As expected for a transcriptional repressor and consistent with the increased H3K4me3 levels, most DEGs were up-regulated. One of these up-regulated genes, *Grin2d*, encodes an NMDAR subunit implicated in neurodevelopmental disorders. We treated mice with the NMDAR antagonist, memantine, and found that this treatment ameliorated sociocommunicative deficits in *Kdm5b* mutant pups. Together, our findings show that KDM5B deficiency results in neurodevelopmental phenotypes, suggest a potential mechanistic link with NMDAR gain-of-function conditions, and suggest that NMDAR antagonists may represent a potential therapeutic avenue for improving sociocommunicative function in individuals with *KDM5B* loss-of-function variants.

The ΔARID mouse model was engineered to delete part of the JmjN and the whole ARID domain to generate a KDM5B protein that lacks demethylase activity (*28*). A recent study confirmed that this so-called ΔARID protein lacks demethylase activity, most likely due to structural protein alterations in the catalytic Jmj domains (*29*). Here, we presented data to suggest that the ΔARID protein is also present at reduced levels in the brain compared to the WT, full-length protein. As previously reported, homozygous ΔARID mice were viable (*28*), and we found no evidence for reduced viability. This contrasts with other homozygous *Kdm5b* loss-of-function mouse models that exhibited pronounced reductions in embryonic or early postnatal viability (*23*, *27*, *28*). Some homozygous null *Kdm5b* mice with an exon 7 deletion, predicted to generate a frameshift and termination mutation, survive to adulthood and exhibited a subtle reduction in social interactions and a deficit in social memory (*23*, *28*).

The initial report of de novo “likely gene disrupting” and missense variants in *KDM5B* in probands with ASD also reported similar variants in the unaffected control population (*12*). It therefore remained unclear whether and to what extent these variants contribute to neurodevelopmental phenotypes. A number of recent studies have now confirmed beyond any doubt that *KDM5B* variants are associated with these neurodevelopmental changes but that these effects are incompletely penetrant. As a consequence, ASD-associated *KDM5B* variants are among the most common inherited ASD-causing variants identified to date (*13*, *22*). The identification of genetic modifiers of *KDM5B* penetrance and expressivity will be of substantial interest. Our findings suggest that variants affecting the NMDAR pathway may be potent modifiers of the social phenotypes.

An improved understanding of KDM5B genotype-phenotype correlations in the context of human neurodevelopmental disorders will be of considerable interest given our findings from the demethylase-deficient mouse strain. A recessive syndrome with compound heterozygosity for two different *KDM5B* variants, which included protein-truncating, splice site, and missense mutations in the JmjC domain, has been reported (*20*, *41*). It remains unclear to what extent a reduction in H3K4me3 demethylation contributes to this condition. Wang *et al.* (*16*) reported an apparent enrichment of heterozygous missense mutations in and around the demethylase domains (JmjN, ARID, and JmjC) of *KDM5B* in individuals with ASD and developmental delay, implicating demethylase activity under these conditions. However, other studies suggest that most of the likely damaging variants in *KDM5B* are found around the plant homeodomain (PHD)2 to PHD3 region. KDM5B interacts with other transcription factors through its PLU domain (*61*) and with histone deacetylase 4 through its N-terminal PHD regions (*62*). Further studies are necessary to decipher the potential impact of different *KDM5B* variants on specific mechanisms, including those that may be independent from its demethylase activity. On the basis of our findings, we propose that variants altering the demethylase activity of KDM5B would primarily affect neurodevelopment postnatally (e.g., via NMDAR signaling), while demethylase-independent variants may affect developmental processes such as neuronal specification and differentiation in the early embryo.

Human patients carrying *KDM5B* variants display a varying degree of brain structural alterations, ranging from a normal MRI to agenesis of corpus callosum, cerebellar vermis alterations, and megalencephaly (*20*, *23*). Notably, one patient showed megalencephaly (*23*), in line with the general increase in absolute brain volumes observed in our *Kdm5b*^Δ*/*Δ^ model. We found increased absolute and relative volumes in fiber tracts such as the corticospinal tract and the anterior commissure. We did not observe alterations specific to the corpus callosum, whereas four individuals with recessive *KDM5B* variants have shown agenesis of the corpus callosum (*41*). This discrepancy suggests that corpus callosum agenesis is not caused by alterations in the demethylase activity of KDM5B, but other, independent KDM5B functions, although genetic background effects are also possible. Three individuals with recessive *KDM5B* variants exhibited altered/unsteady gait (*23*). This phenotype could be related to the structural changes we observe in the cerebellum in *Kdm5b*^Δ*/*Δ^ animals. The thalamus and brain stem, two crucial areas controlling posture and gait, also showed increased absolute volumes in *Kdm5b*^Δ*/*Δ^ homozygous mice. The aberrant size of the striatum is in line with the reduced performance of *Kdm5b*^Δ*/*Δ^ mice in the accelerating rotarod task (*63*). De novo, likely gene-disrupting variants in *KDM5B* have also been linked to primary motor stereotypies, another phenotype associated with alterations in the cerebellum (*64*) and cortico-striatal-thalamo-cortical pathways (*60*, *65*–*67*). Abnormalities in these brain regions and pathways could underlie the repetitive behaviors observed in *Kdm5b*^Δ*/*Δ^ mice. Further work, by for example comparing neuronal activation during USVs in WT and mutant pups, are necessary to identify the regional and cell type abnormalities ultimately responsible for the behavioral phenotypes.

Chromatin modifiers and remodelers typically regulate hundreds of gene loci throughout the genome and function in multiple cell types and at different stages of development. For instance, in the case of CHD8, many ASD-associated genes are dysregulated in the *Chd8*-deficient brain, making it difficult to determine the exact downstream genes responsible for neurodevelopmental phenotypes (*68*–*73*). Our snRNA-seq analysis suggests that KDM5B functions in multiple cell types in the developing postnatal brain, including neuronal progenitors, excitatory and inhibitory neurons, and vascular cells. Our H3K4me3 CUT&Tag-seq experiment identified potential direct targets of KDM5B in the PND5 neocortex, including *Grin2d*. However, attempts to directly demonstrate KDM5B recruitment to the *Grin2d* promoter by chromatin immunoprecipitation were unsuccessful due to the quality of commercially available antibodies.

Given the potential complexities of KDM5B function in the developing brain, we were surprised to find that treatment with an NMDAR antagonist was sufficient to improve some for the ASD-associated behavioral phenotypes in *Kdm5b* mutants. Memantine is a noncompetitive antagonist of NMDARs with low affinity and rapid kinetics (*74*). This treatment presumably normalizes an abnormal gain in NMDAR function due to *Grin2d* up-regulation in the early postnatal brain. *Grin2d* expression in rodents starts at late embryonic stages, peaking at PND7 (*75*). In the hippocampus, NMDAR2D-containing receptors mediate synaptic transmission in interneurons (*76*), and they constrain short-term and long-term potentiation in area CA1 (*77*). Our attempts to measure AMPA:NMDA ratios in PND5 hippocampus were unsuccessful due to the preponderance of silent synapses that lack AMPAR in the PND5 neocortex (*50*, *51*). Further work is therefore required to determine the exact mechanism whereby *Grin2d* overexpression and memantine treatment affect sociocommunicative behaviors in the mouse model.

In humans, gain-of-function mutations in the NMDAR subunit gene *GRIN2D* are associated with GRIN2D-related developmental and epileptic encephalopathy, a condition associated with developmental delay, intractable seizures, abnormal muscle tone, movement disorders, and ASD (*45*, *78*–*80*). NMDAR antagonists such as memantine and ketamine have proven therapeutically useful for treatment of epileptic encephalopathies associated with increased NMDAR function (*45*). In addition to hypotonia and ASD, two individuals carrying *KDM5B* variants show epileptic spasms and generalized seizures (*23*, *81*). Future studies assessing the effects of different *Kdm5b* loss-of-function variants on excitatory neurotransmission will be of interest, especially if these can be combined with NMDAR antagonist treatment.

Memantine treatment has been evaluated in individuals with ASD, with varied effects, not unexpected given that both increased and reduced NMDAR function have been implicated in ASD (*30*, *31*, *82*–*86*). The present study, together with studies on *Shank2*-deficient and VPA rodent models (*34*, *38*), suggests that treatment with NMDAR antagonists is likely to benefit individuals where symptoms are caused by increased NMDAR function.

In addition to *Grin2d*, the other differentially expressed ASD-associated gene worth noting is *Mcm6*. *Mcm6* was the most down-regulated gene in *Kdm5b* mutants. De novo *MCM6* variants were first detected in ASD probands by Iossifov *et al.* (*12*), with further reports by Takata *et al.* (*42*). Subsequently, missense mutations in *MCM6* were associated with neurodevelopmental phenotypes that include ASD, delayed speech development, and epilepsy by Smits *et al.* (*87*). MCM6 is important for primary ciliogenesis and cell proliferation (*87*, *88*), two crucial processes during brain development (*89*).

Limitations of our study include the difficulty in distinguishing between the contribution of H3K4me3 demethylase deficiency or reduced protein levels on the observed phenotypes. The *Kdm5b*^Δ*ARID*^ allele represents a hypomorphic allele that lacks demethylase activity and an apparent reduction in steady-state protein levels. Regardless, we do find a robust up-regulation of steady-state H3K4me3 levels in the neocortex at the promoters of up-regulated genes, indicating that H3K4me3 demethylase activity is indeed affected in these mice. Although we were unable to directly measure changes in NMDAR signaling at early postnatal stages due to technical limitations, our treatment studies with memantine support the idea that increased NMDAR signaling is responsible for the USV deficits. Future work using more selective NMDAR antagonists and genetic rescue experiments are needed to further support our model and assess the translational potential of targeting NMDAR signaling in the context of KDM5B neurodevelopmental disorders.

In conclusion, we have found that a deficiency in the KDM5B demethylase results in ASD-associated behavioral phenotypes in mice. *KDM5B* deficiency was associated with increased levels of H3K4me3, demonstrating that KDM5B functions as an H3K4me3 demethylase in the developing brain. These alterations affected gene expression, with most DEGs up-regulated, confirming that KDM5B acts primarily as a transcriptional repressor. Last, we provide evidence to suggest that abnormal elevation of NMDAR2D-containing NMDAR signaling in the postnatal brain is responsible for the sociocommunicative deficits in these mice. These observations functionally link *KDM5B* deficiency with childhood neurodevelopmental disorders caused by NMDAR gain-of-function mutations and suggest a potential therapy to alleviate some of the behavioral dysfunction associated with *KDM5B* deficiency.

## MATERIALS AND METHODS

### Animals

The *Kdm5b*^Δ*ARID*^ mouse line has been described (*28*). In this mouse line, *Kdm5b* exons 2, 3, and 4 were removed, leading to the deletion of the entire ARID domain and the truncation of the carboxyl end of the JmJ domain. Mice were maintained in a C57BL/6J background, and experimental animals were produced by *Kdm5b*^+/ΔARID^ × *Kdm5b*^+/ΔARID^ intercrosses. Animals were housed in ventilated cages (37 × 20 × 16 cm; Techniplas UK Ltd., Leicester, UK) with ad libitum access to water and food (LabDiet PicoLab rodent irradiated, #5R53) and kept at 19° to 22°C and 40 to 60% humidity, under a 12:12-hour light/dark cycle. The cages contained sawdust (Lignocel wood fiber) and nesting material. A maximum of five animals was housed in the same cage. Our study examined male and female animals, and similar findings are reported for both sexes. All animal procedures were approved by King’s College London Animal Welfare and Ethical Review Body and the UK Home Office (Home Office Project licenses P8DC5B496 and PP6246123).

### Genotyping of mice

For genotyping, genomic DNA was extracted from ear notches using Proteinase K digestion or the HotSHOT method (*90*). Genotyping was performed by PCR as described (*91*). WT primers (forward, 5′-CCTTAGACGCAGACAGCACA-3′; reverse, 5′-CGTGTTTGGGCCTAAATGTC-3′) yielded a WT band of 275 base pairs (bp). KDM5B-ΔARID primers (forward, 5′- TGCTCCTGCCGAGAAAGTATCC-3′; reverse, 5′-CCACCCCCCAGAATAGAATGA-3′) yielded a mutant band of 663 bp. The following thermal cycles steps were used for the genotyping reactions: 95°C, 2 min; 35 cycles × (95°C, 15 s; 64°C, 15 s; 72°C, 15 s); 72°C, 12 min.

### Western blots

#### KDM5B and histone blots

Brain cortices and hippocampi were dissected from PND5 pups, and whole-cell protein was prepared by lysing in 8 M urea, 1% CHAPS, and 50 mM tris (pH 7.9) lysis buffer containing protease inhibitors. Samples were rotated for 30 min at 4°C and then centrifuged for 60 min to remove DNA. Supernatant was stored at −80°C. All reagents and equipment were from Bio-Rad unless stated otherwise. Samples were prepared with Laemmli buffer containing 10% β-mercaptoethanol and resolved with Mini-PROTEAN precast polyacrylamide gels (7.5 and 4 to 15% gels to analyze proteins with high and medium/low molecular weights, respectively) and tris/glycine/SDS buffer. Proteins were transferred to a nitrocellulose membrane with the Trans-Blot turbo system. Membranes were blocked with 5% nonfat dry milk (Cell Signaling Technology) or 5% bovine serum albumin (BSA; Sigma-Aldrich) diluted in tris-buffered saline containing 0.1% Tween 20 (TBS-T), followed by incubation overnight at 4°C with primary antibodies diluted in 5% BSA in TBS-T. Membranes were washed 3 × 10 min in TBS-T before incubation for 1 hour at room temperature with secondary antibodies diluted in TBS-T containing 5% nonfat dry milk. After washing 3 × 10 min in TBS-T, proteins were detected with Clarity ECL reagent, and membranes were imaged using the ChemiDoc system. Densitometric analyses were performed with the ImageJ software (National Institutes of Health). The following antibodies were used (1:1000 dilution unless stated otherwise): anti-KDM5B (Abcam, #181089), anti-H3K4me3 (Cell Signaling Technology, #9751), anti–total H3 (Cell Signaling Technology, #9715), HSP90 (Santa Cruz Biotechnology, sc13119), actin (1:5000; Abcam, #ab8227), goat anti-rabbit and anti-mouse horseradish peroxidase secondary antibodies (1:5000; Thermo Fisher Scientific, #31460 and Proteintech, #SA00001-1, respectively). Uncropped Western blot images are shown in fig. S8.

#### NMDAR2D Western blots

Brain cortices were dissected from PND5 pups, and synaptosomes were isolated using Syn-PER Synaptic Protein Extraction Reagent (Thermo Fisher Scientific) following the manufacturer’s protocol. The synaptosome pellet was resuspended in radioimmunoprecipitation assay buffer containing protease inhibitors, and the protein concentration was quantified using a bicinchoninic acid (BCA) assay (Thermo Fisher Scientific). Laemmli buffer (Bio-Rad) with DDT was added to 15 μg of each sample and loaded into a 4 to 20% precast polyacrylamide gel (Bio-Rad, 4561095). The gel was electrophoresed for 10 min at 50 V and then 1 hour at 120 V in tris-gycine SDS buffer (Bio-Rad). The gel and nitrocellulose membrane were then equilibrised in transfer buffer (Bio-Rad) for 10 min before being transferred for 1 hour at 80 V in the transfer buffer. The membranes were stained using Ponceau S (Merck) to check for protein transfer and blocked in phosphate-buffered saline with Tween-20 (PBS-T) containing 5% nonfat milk buffer. The membrane was incubated with the primary antibodies overnight at 4°C and then repeatedly washed with PBS-T before incubation in secondary antibodies for GRIN2D (Merck, MAB5578; 1:500) and β-actin (Merck, A5316; 1:5000) overnight at 4°C. The membranes were repeatedly washed with PBS-T before incubating them in DyLight 800 secondary antibody (Thermo Fisher Scientific, SA5-10164; 1:5000) for 1 hour in the dark at room temperature. The membranes were then washed three times and imaged and analyzed on the LICOR Odyssey CLx machine. Uncropped full scans of the Western blot membranes can be found in fig. S8.

### Behavior

Different batches of mice were used for recording pup USVs, for adult behavior testing, and for the treatment with memantine (see Figs. 2A and 8C). Adult tests were carried out in the following order: open field, EPM, reciprocal social tests, marble burying, three-chamber social approach, and rotarod as previously described (*68*, *70*). Behavioral experiments were conducted between 8:00 and 18:30 under standard room lighting conditions. Cages were changed every other week but at least 24 hours before running a behavioral experiment. Animal tracking was performed with EthoVision (Noldus Information Technologies B.V., Wageningen, The Netherlands). After each behavioral trial, fecal boli and urine were removed, and surfaces were cleaned with 1% Anistel solution (high-level surface disinfectant, Tristel Solution Ltd., Cambridgeshire, UK) to remove any odors. Littermates served as controls, and each experiment included animals from different litters. Mice were assigned to experimental groups using block randomization under blinded conditions.

#### Ultrasonic vocalizations

USVs were recorded in pups across 3-min sessions in response to social separation from the mother and siblings at PND2, PND4, PND6, PND8 and PND12 as described (*92*)*.* The tattooing for early identification of the mice was carried out on PND1-2. Tattooing (animal tattoo ink green paste, Ketchum, #KI1471) for identification was carried out by inserting the ink subcutaneously through a 0.3-mm hypodermic needle into the center of the paw. Tattooing was carried out at PND2 in the experiment described in Fig. 2 and at PND1 for the rescue experiment. USV testing as in Fig. 2 was performed on a batch of 22 mice (males: 3 WT and 6 mutant; females: 7 WT and 6 mutant). The experiment included in Fig. 8 was performed on a batch of 15 mice (4 male and 3 female saline and 5 male and 2 female memantine). During testing, each pup was transferred from its homecage to an empty glass container in a sound-attenuating box placed in a different room. Lights were turned off during the USV recording. An Ultrasound Microphone (Avisoft UltraSoundGate condenser microphone capsule CM16, Avisoft Bioacoustics, Berlin, Germany), sensitive to frequencies of 10 to 180 kHz, was placed through a hole in the middle of the cover of the sound-attenuating box, about 20 cm above the pup in its glass’ container. USVs were recorded using the Avisoft Recorder software (version 3.2). The glass container was cleaned with 10% ethanol between pups and with 70% ethanol between litters. For acoustical analysis, recordings were transferred to Avisoft SASLab Pro (version 4.40), and a fast Fourier transformation was conducted. Spectrograms were generated at a frequency resolution of 488 Hz and a time resolution of 1 ms.

### Memantine treatment

A memantine (Cayman Chemical Company, #14184) solution was prepared fresh in saline (0.56 mg/ml for early postnatal and 1 mg/ml for adult mice) each day. A total of 10 μl/g of saline or memantine solution was administered intraperitoneally 30 min before recording USVs or running the marble burying and accelerating rotarod tests. Doses were 5.6 mg/kg for USVs (*93*) and 10 mg/kg for the marble burying and accelerating rotarod tests (*54*, *55*).

### MRI

#### Perfusions

Mice were anesthetized with pentobarbital and intracardially perfused with 30 ml of 0.1 M PBS containing heparin (0.05 U/ml; Sigma-Aldrich) and 2 mM ProHance (Bracco Diagnostics, a Gadolinium contrast agent), followed by 30 ml of 4% paraformaldehyde (PFA) containing 2 mM ProHance (*68*, *94*). Perfusions were performed with a minipump at a rate of ~1 ml/min. After perfusion, mice were decapitated, and the skin, lower jaw, ears, and the cartilaginous nose tip were removed. The brain and remaining skull structures were incubated in 4% PFA + 2 mM ProHance overnight at 4°C and then transferred to 0.1 M PBS containing 2 mM ProHance and 0.02% sodium azide for at least 1 month before MRI scanning (*95*).

#### MRI (ex vivo)

A 7-T 306-mm horizontal bore magnet (BioSpec 70/30 USR, Bruker, Ettlingen, Germany) with a ParaVision 6.0.1 console was used to image brains in skulls. Eight samples were imaged in parallel using a custom-built 8-coil solenoid array. To acquire anatomical images, the following scan parameters were used: T2-weighted three-dimensional fast spin echo sequence with a cylindrical acquisition of k-space, repetition time/echo time/echo train length (TR/TE/ETL) = 350 ms/12 ms/6, *TE*_eff_ = 30 ms, four effective averages, filed of view/matrix-size = 20.2 by 20.2 by 25.2 mm/504 by 504 by 630, total imaging time = 13.2 hours. The resulting anatomical images had an isotropic resolution of 40-μm voxels.

#### Imaging registration and analysis

Image registration is necessary for quantifying the anatomical differences across images. The registration consisted of both linear (rigid then affine) transformations and nonlinear transformations. These registrations were performed with a combination of mni_autoreg tools (Collins, 1994) and Advanced Normalization Tools (*96*). After registration, all scans were resampled with the appropriate transform and averaged to create a population atlas representing the average anatomy of the study sample. The results of these registrations were deformation fields that transform images to a consensus average. Therefore, these deformations fields quantify anatomical differences between images. As detailed in previous studies (*97*, *98*), the Jacobian determinants of the deformation fields were computed and analyzed to measure the volume differences between subjects at every voxel. A preexisting classified MRI atlas was warped onto the population atlas (containing 282 different segmented structures encompassing cortical lobes, large white matter structures such as the corpus callosum, ventricles, cerebellum, brain stem, and olfactory bulbs to compute the volume of brain structures in all the input images) (*99*–*103*). A linear model with a genotype predictor was used to assess significance. The model was either fit to the volume of every structure independently (structure-wise statistics) or fit to every voxel independently (voxel-wise statistics), and multiple comparisons in this study were controlled for using the FDR (*104*).

### RNA sequencing

Total RNA was prepared as described (*68*). RNA quality was analyzed using Agilent Total RNA 6000 Pico on a Bioanalyser (Agilent, 2100). Pair-end sequencing (150-bp read length) was performed on the Illumina HiSeq 6000 platform. Further data analyses were performed using the Galaxy Europe server (https://usegalaxy.eu). Quality of the raw data was checked using FastQC version 0.11.9. Reads were aligned to the mouse genome (mm10) using RNA STAR Galaxy Version 2.7.8a, and aligned reads were counted using featureCounts Galaxy version 2.0.1. Differential expression analyses were performed using DESeq2 Galaxy version 2.11.40.7. Heatmaps were generated with the R package pheatmap. Volcano plots were generated in GraphPad Prism 9.4.1. (*105*). The criteria to select DEGs were FDR < 0.05 and |log_2_ fold change| > 0.263. Gene set enrichment analyses were performed on R with clusterProfiler as described in https://learn.gencore.bio.nyu.edu/rna-seq-analysis/gene-set-enrichment-analysis (accessed September 2024). Venn diagrams were created to show the extent of overlap between heterozygous and homozygous DEGs (FDR < 0.05), and ASD-associated genes obtained from the SFARI Gene database (https://gene.sfari.org/database/human-gene/, accessed October 2025; see table S13) or ID-associated genes obtained from the Genomics England PanelApp intellectual disability database (https://panelapp.genomicsengland.co.uk/panels/285/, version 4.42, accessed December 2022; see table S14).

### Nuclei isolation for CUT&Tag and snRNA-seq

Nuclei were isolated from the cortex of PND5 pups by incubating tissue for 15 min on ice in lysis buffer [10 mM tris-HCL (pH 7.4), 10 mM NaCl, 3 mM MgCl_2_, and 0.1% NP-40 substitute]. Tissue was mechanically homogenized with a 27-gauge needle before layering on top of 1.8 M sucrose solution. A total of 1.7 μM dithiothreitol was added to the sucrose solution for snRNA-seq nuclear extractions. Samples were centrifuged at ×13,000*g* for 45 min at 4°C. The resulting nuclear pellet was washed and resuspended in 2% BSA in PBS. Extracted nuclei were counted manually and checked for quality using trypan blue. For snRNA-seq, 200 U RiboLock RNase inhibitor (Thermo Fisher Scientific) was added to lysis buffer, wash buffer, and resuspension buffer.

### CUT&Tag sequencing

CUT&Tag was performed using the EpiCypher CUTANA CUT&Tag kit as per manufacturer’s instructions (EpiCypher, v2). A total of 100,000 nuclei, previously stored in BAMBANKER at −80°C, were used per reaction. In short, ConA beads were activated and bound to the isolated nuclei. A total of 2 μl of stock K-MetStat Panel (EpiCypher, 19-1002) was added to each reaction before addition of the antibodies. A total of 0.5 μg of anti-H3K4me3 (EpiCypher, 13-0060) or rabbit immunoglobulin G negative control (EpiCypher, 13-0042) primary antibodies was added to each reaction and incubated on a nutator at 4°C overnight. Nuclear slurry was incubated with anti-rabbit secondary antibody (EpiCypher, 13-0047) at room temperature for 30 min. Tagmentation was carried out for 1 hour at 37°C. Libraries were prepared by PCR with a unique pair of i5 and i7 indexing primers with the following parameters: 58°C, 5 min; 72°C, 5 min; 98°C, 45 s; 98°C, 15 s, 60°C, 10 s, 16 cycles; 72°C, 1 min; 4°C hold. Library quality was assessed using the TapeStation (Agilent) with D1000 ScreenTape and reagents. Libraries were quantified using the Qubit fluorometer with 1× double-stranded DNA high sensitivity assay kit. Paired-end (150 bp) sequencing was performed on the Illumina NovaSeq X.

The workflow for CUT&Tag-seq data analysis was broadly performed using an adapted nf-core/cutandrun Nextflow pipeline (*106*, *107*). Briefly, FASTQ files were first checked for sequencing quality using FastQC, followed by trimming of adapter and low-quality sequences by Trim Galore! (version 0.6.5). Paired-end reads were aligned to the GRCm38 (mm10) genome using Bowtie2 (version 2.3.5.1) (*108*). SAMtools (version 1.22.1) (*109*) was then used to sort and index BAM files, followed by marking of duplicate reads by Picard (version 3.4.0). BedGraph files were generated using bedtools (2.31.0), and bigWig files were generated using bedGraphToBigWig (version 4.8.2) and visualized on the Integrative Genomics Viewer (IGV). Peak calling was performed either using MACS2 (version 2.2.9.1) or SEACR (version 1.3) (*110*) and then merged to generate consensus peaks using bedTools. To spike-in normalize files for analysis and visualization, deepTools (version 3.5.6) (*111*) was used to apply scaling factors calculated from SNAP-CUTANA K-MetStat Panel spike-ins. A scaling factor was calculated for each sample by dividing the percentage of total GRCm38-aligned reads by the percentage of total reads taken up by spike-in barcodes—this scaling factor was then applied to each sample. Following this, deepTools computeMatrix was then used to determine enrichment around gene TSS and visualized using plotHeatmap. Comparisons of H3K4me3 enrichment at gene TSS between samples were performed using DiffBind (version 3.21) using windows of ±1 kb around the TSS.

### Parse sequencing

Neocortical brain samples from eight WT and eight mutant mice were dissected out and stored (−80°C) until sample processing for nuclei isolation. Isolated nuclei were immediately fixed using the Evercode Fixation kit (Parse Biosciences). Sequencing libraries were generated using the Evercode WT kit (chemistry version 3, Parse Bioscience). Paired-end sequencing (150 bp) was performed using the Illumina NovaSeq X. Raw sequencing reads were processed with the Trailmaker pipeline (cloud-based data processing platform from Parse Biosciences) using default settings to demultiplex sublibraries, align reads to the GRCm39 reference genome, and produce cell-by-gene count matrices. Downstream analyses were completed with Scanpy (v.1.11.0). Quality control was performed using standard parameters based on guidelines published previously (*112*). Briefly, we filtered out cells expressing a low or too high number of genes and total transcripts counts and those with a high mitochondrial proportion. We further used Scrublet [pp.scrublet() function] to remove doublets (*113*). Data were then normalized and log transformed using Scanpy’s pp.normalize_total() and pp.log1p() functions. Log-transformed counts were then used to calculate highly variable genes. Integration was performed using Scanpy’s external pp.harmony_integrate() function using the different samples as a covariate. We then used Harmony integrated principal components analysis for dimension reduction and unsupervised Leiden clustering of the data with a final resolution of 0.5 to find clusters. To visualize the data, we used uniform manifold approximation and projection (UMAP). Cluster annotation was performed manually based on DEGs in each cluster and previous literature (*114*, *115*).

### Statistics

Data are reported as mean ± SEM. Graphs show individual data points. Normal distribution was tested with D’Agostino and Pearson omnibus, Shapiro-Wilk, and Kolmogorov-Smirnov normality tests. If the test was passed, then statistical analysis was performed using parametric statistical analyses. Before pairs of comparisons, we performed the *F* test to compare variances. Statistical analyses were performed using the unpaired two-sided Student’s *t* test and the three- and two-way ANOVA. Only when the interaction effect was significant (*P* < 0.05) was these followed with the appropriate post hoc tests as indicated in the figure legends. *t* test with Welch’s correction was applied when variances were unequal. Significant *P* values (*P* < 0.05) are reported in Results, and/or figure legends provide details of relevant statistical parameters, including group sizes. Statistical analyses were performed with GraphPad Prism (version 9.4.1). Researchers were blinded, and in vivo studies were randomized by blocks of animals. Source data for the different figures are provided in table S12.
