# Supplementary material for: Autism-like phenotypes and increased NMDAR2D expression in mice with KDM5B histone lysine demethylase deficiency
Source: Sci Adv. 2026 May 20;12(21):eadq6577. doi: 10.1126/sciadv.adq6577 (PMC13189110; doi:10.1126/sciadv.adq6577)
Supplement: Supplementary file 1 — Supplementary Methods Figs. S1 to S8 Legends for tables S1 to S14 [file sciadv.adq6577_sm.pdf]

Science Advances
AAAS

# Supplementary Materials for

## Autism-like phenotypes and increased NMDAR2D expression in mice with KDM5B histone lysine demethylase deficiency

Leticia Pérez-Sisqués *et al.*

Corresponding author: M. Albert Basson, m.a.basson@exeter.ac.uk

*Sci. Adv.* **12**, eadq6577 (2026)
DOI: 10.1126/sciadv.adq6577

**The PDF file includes:**

Supplementary Methods
Figs. S1 to S8
Legends for tables S1 to S14

**Other Supplementary Material for this manuscript includes the following:**

Tables S1 to S14

**Supplementary methods**

*qRT-PCR analysis*

cDNA was synthesised from 200-400ng RNA with UltraScript 2.0 cDNA Synthesis kit (PCR Biosystems) according to the manufacturer's instructions. qRT-PCRs were performed on a BioRad CFX384 using qPCRBIO SyGreen Mix Lo-ROX (PCR Biosystems). See Table S9 for primer sequences. Relative expression levels were calculated using the $2^{-\Delta\Delta CT}$ method and *Hprt* was used as endogenous control gene.

*Measurement of AMPA/NMDA ratios*

Mice were anesthetized by isoflurane inhalation before decapitation. Brains remained in ice-cold cutting solution (in mM: 240 sucrose, 5 KCl, 1.25 $Na_2PO_4$, 2 $MgSO_4$, 1 $CaCl_2$, 26 $NaHCO_3$ and 10 D-glucose) equilibrated with 95% $O_2$/5% $CO_2$ throughout the dissection and slicing procedures. 350µm-thick sagittal brain slices were obtained with a 7000smz-2 vibratome (Campden instruments). Slices were allowed to recover in artificial cerebrospinal fluid (aCSF, in mM: 124 NaCl, 5 KCl, 1.25 $Na_2HPO_4$, 2 $MgSO_4$, 2 $CaCl_2$, 26 $NaHCO_3$ and 20 D-glucose) equilibrated with 95% $O_2$/5% $CO_2$ for 1h at 32° before recording.

Whole-cell patch clamp recordings were taken from CA1 pyramidal neurons. Slices were continuously perfused with aCSF with 10µM gabazine equilibrated with 95% $O_2$/5% $CO_2$, held in position using a platinum wire harp and visualised using a Nikon Eclipse FN1 microscope and a Rolera Bolt camera under a 40x water-dipping objective. All recordings were performed at room temperature using a Patch clamp EPC 10 USB amplifier and PatchMaster software (HEKA) with signals filtered at 10 kHz and sampled at 50 kHz. All traces were recorded and analyzed blind to genotype.

The experiment was performed using a $Cs^{2+}$-$MeSO_4$-loaded internal solution (in mM: 130 $CsMeSO_4$, 4 $MgCl_2$*6$H_2O$, 0.2 EGTA, 10 HEPES, 4 Na2ATP, 0.4 Na3GTP, 10 $Na^+$-

phosphocreatine, 1 QX-314). A parallel bipolar electrode connected to a DS3 constant current isolated stimulator (Digitimer) was placed 150µm apart (dorso-ventral and anterior-porterior) from the recording area to target the Schaffer collaterals. One minute after breaking in, a square voltage-step pulse (± 10 mV for 10ms) was recorded before and after each trace to determine series resistance. Any cell determined to have series resistance values > 20 MΩ or whose series resistance varied by >20% over the course of recording, were excluded from further analysis. Cells were initially clamped at -70mV to measure maximal AMPAR-mediated currents. The magnitude of the stimulation pulse was determined for each cell. For that purpose, 40µs depolarising stimuli were administered to determine the minimum amplitude to evoke a EPSC of 50-200pA of amplitude. The AMPA component was then recorded. Following a 1-minute recovery period, cells were then held at +40mV to measure the NMDAR response. Both currents were recorded in 10 separate sweeps (0.1Hz), each one consisting of a single 40µs depolarising stimuli. The amplitudes of the peak of the evoked currents were determined using a custom-written MATLAB script. The AMPA/NMDA ratio was calculated for each cell as (average of -70mV responses) / (average of +40mV responses).

## Supplementary figures

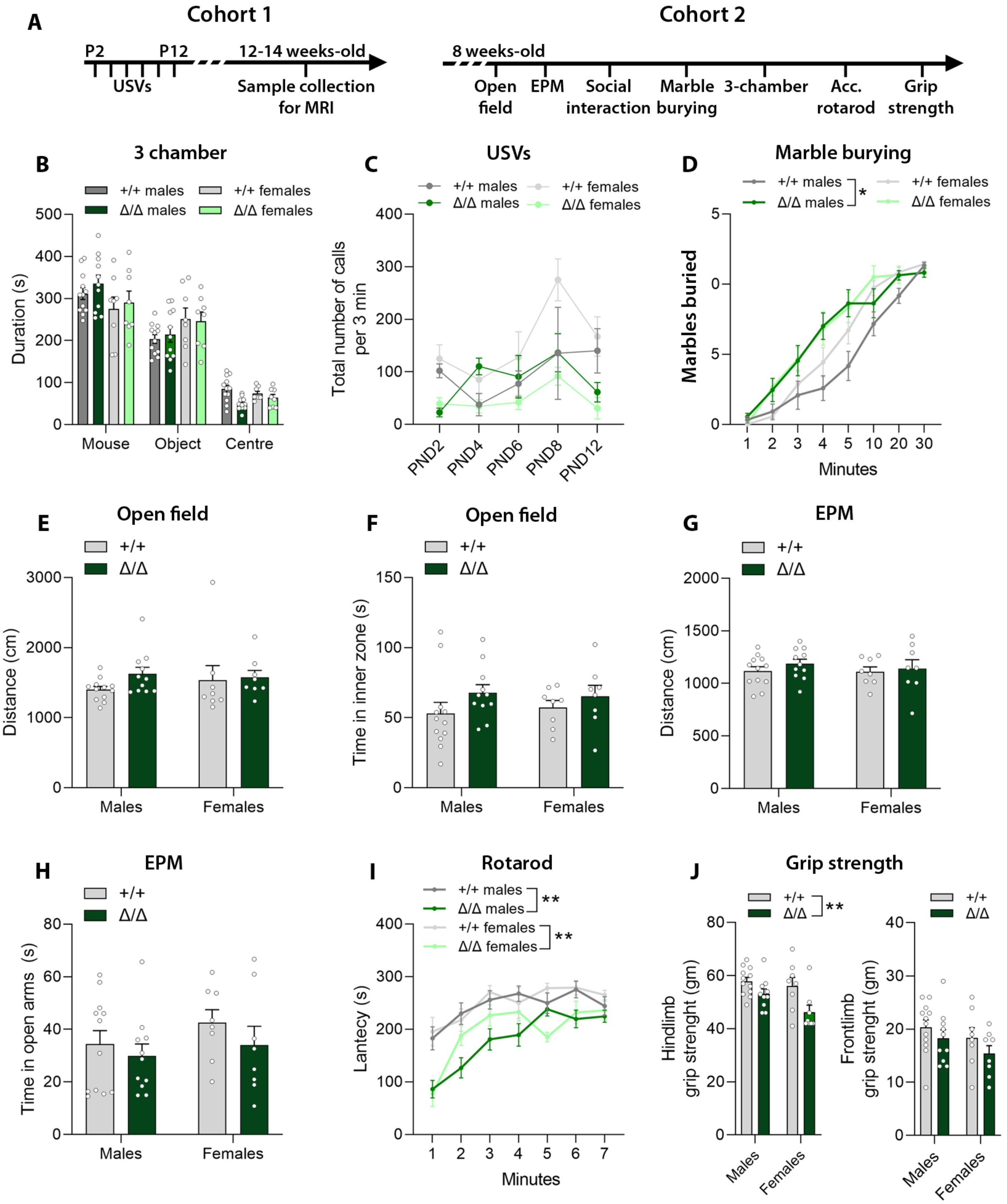


**Fig. S1. Behavioural assessment of *Kdm5b* ΔARID homozygous mice.** Behavioural assessment of a cohort of adult mice (A, C-I) (+/+ n=11-12 males and 7-8 females; Δ/Δ n=10-11 males and 6-8 females) and pups (B) (+/+ n=3 males and 7 females; Δ/Δ n=6 males and 6 females). **A)** Experimental time-course. Note that different cohorts were used for experiments with neonatal and adult mice. **B)** Time spent, in seconds, in each chamber in the 3 chamber sociability test, for

male and female animals. Three-way ANOVA sex effect: $F_{(1,105)}$=2.49e-005, p=0.9960. **C)** Total number of ultrasonic vocalisations (USVs) during 3 minutes testing sessions on the indicated postnatal days. Three-way ANOVA sex effect: $F_{(1,18)}$=0.3555, p=0.5584. **D)** Number of marbles buried during a 5 minutes test. Three-way ANOVA sex effect: $F_{(1,3160)}$=0.9021, p=0.3437. $ p<0.05 mutant male versus WT male. **E)** Distance travelled in the outer area of an open field arena during a 5 minute test. Two-way ANOVA sex effect: $F_{(1,35)}$=0.1520, p=0.6990. **F)** Time spent in the inner zone of the arena during the open field test. Two-way ANOVA sex effect: $F_{(1,35)}$=0.01616, p=0.8996. **G,H)** Distance travelled (F) and time spent (G) in in the open arms of the elevated plus maze (EPM) is shown. Two-way ANOVA sex effect: $F_{(1,35)}$=0.2334, p=0.6320 and $F_{(1,35)}$=1.263, p=0.2688, respectively. **I)** Mean latency of mice to fall from the rotarod during 7 trials in one day. Three-way ANOVA sex effect: $F_{(1,35)}$=0.7865, p=0.3812. **J)** Hind and frontlimb grip strength. Data is shown as mean ±SEM and was analysed with Two-way ANOVA (A, D-G, I) followed by Tukey's multiple comparisons test or repeated measures ANOVA (B, C, H) followed by Sidak's post hoc test. Sex differences were assessed with three-way ANOVA (A-C, H). *$P$<0.05,

**$P$<0.01.

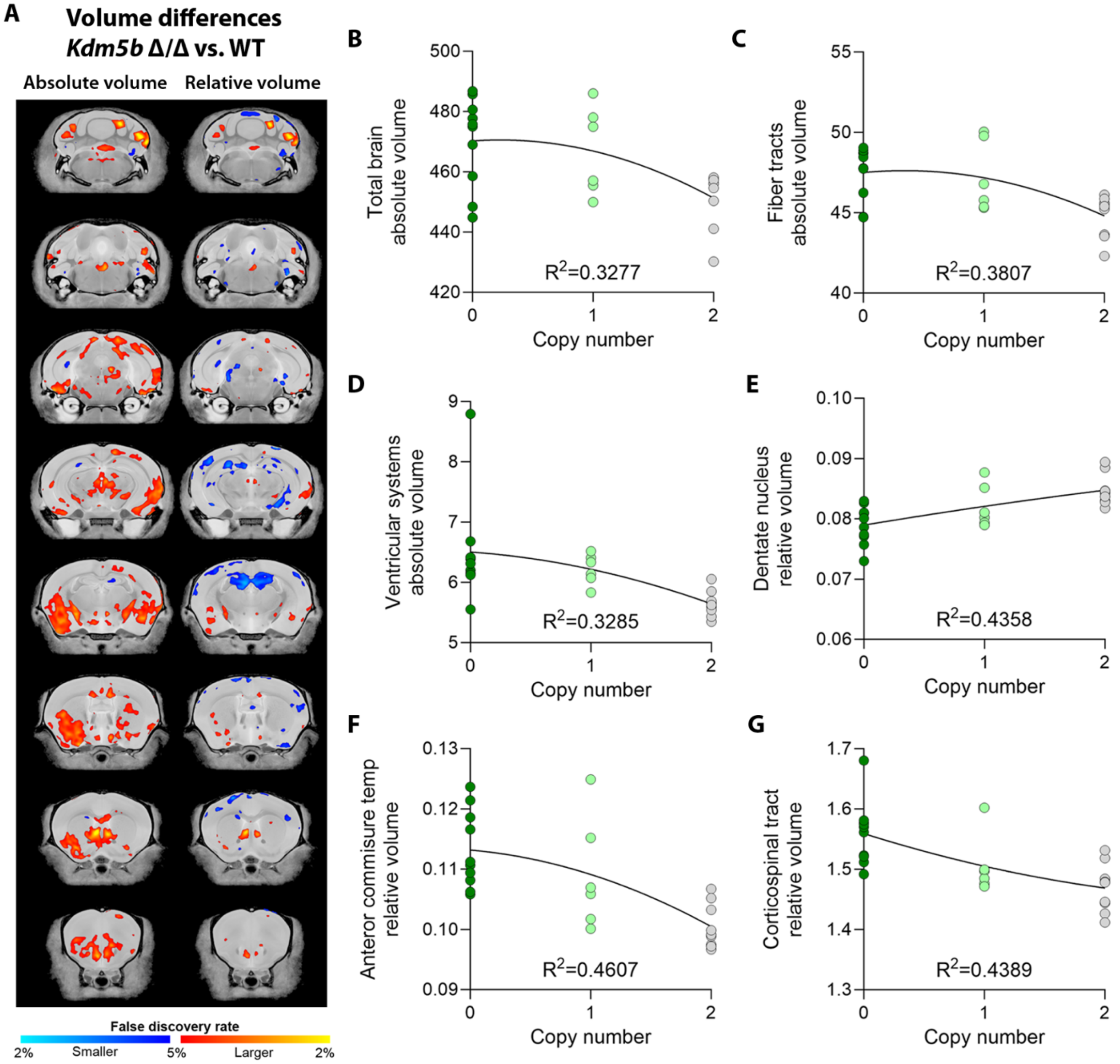


**Fig. S2. Structural MRI. A)** Voxel-wise differences in volume (absolute, left; relative, right) between wild-type and *Kdm5b*$^{+/\Delta}$ mutant littermates. **B-G)** Nonlinear (polynomial) correlation analysis between absolute and relative volumes and gene copy number. Each point represents an individual animal. Nod. lobe, cerebellar nodular lobe; Thal., thalamus; Cort. tract, corticospinal tract; Str., striatum; Pall., pallidum; Lat. Sep., lateral septum; Taenia tc., taenia tecta; Ant. olf., anterior olfactory nucleus.

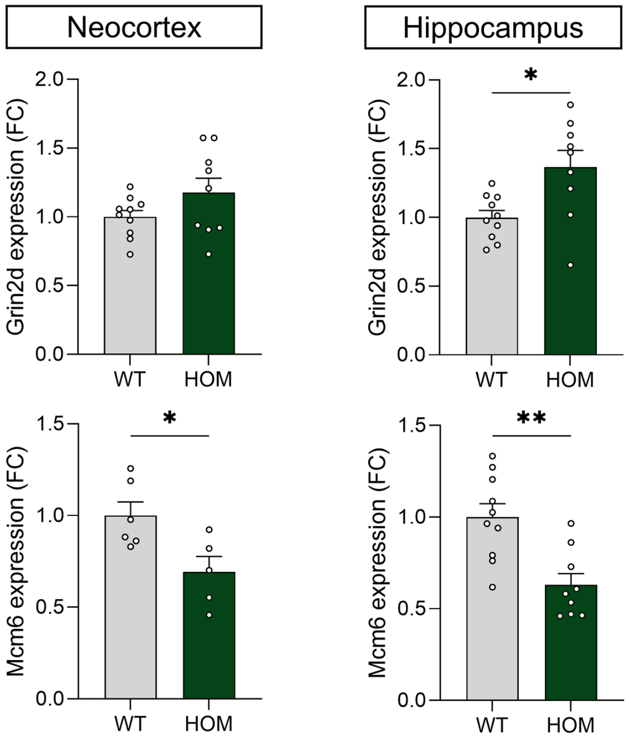


**Fig. S3. qRT-PCR validation of bulk RNA-seq data.** qRT-PCR analysis of *Mcm6* and *Grin2d*, relative to *Hprt*, from total RNA extracted from the cortex (first column) or hippocampus (second column) from P5 mouse brains. Student's t-test. *$P$<0.05, **$P$<0.01. N=8 males +/+, 8 males Δ/Δ, 6 females +/+ and 5 females Δ/Δ.

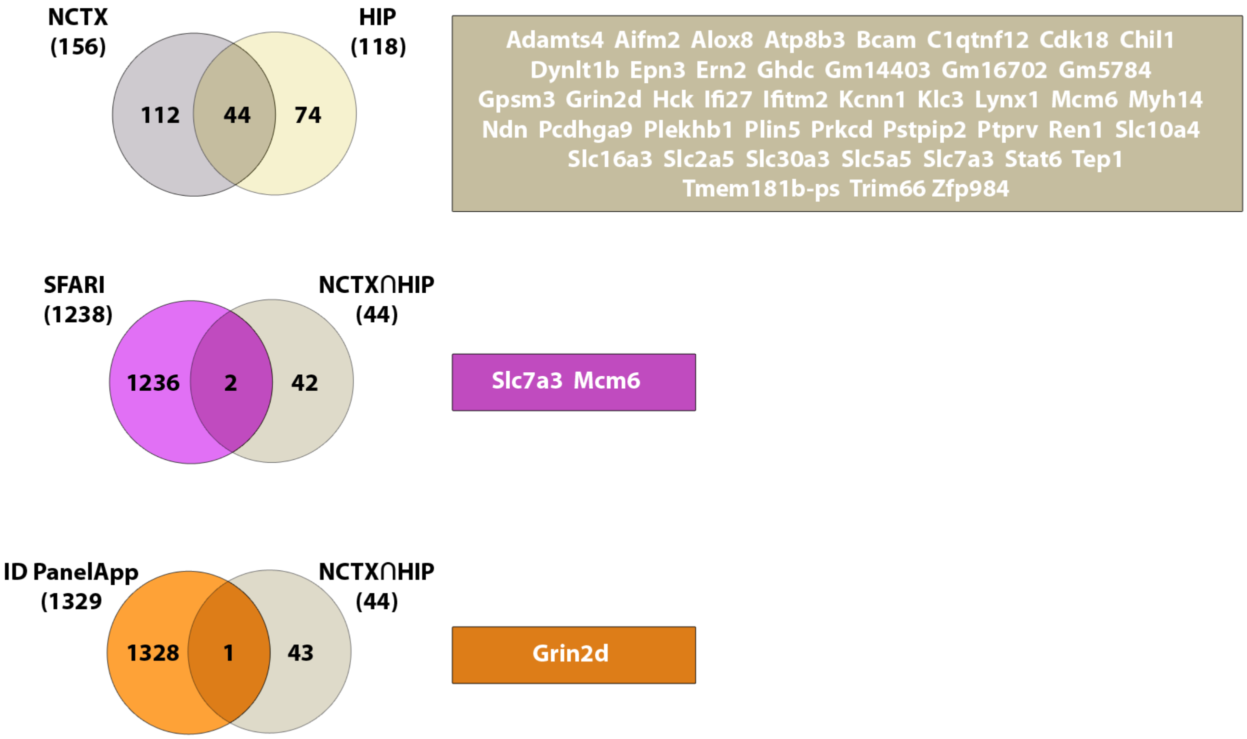


**Fig. S4. DEG overlap between neocortex and hippocampus.** Venn diagrams showing the extent of overlap of the DEGs in the neocortex (NCTX) and hippocampus (HIP), together with the overlap between those overlapping genes in the neocortex and hippocampus (NCTX∩HIP) and the SFARI and ID PanelApp databases. Overlapping genes are shown on the right.

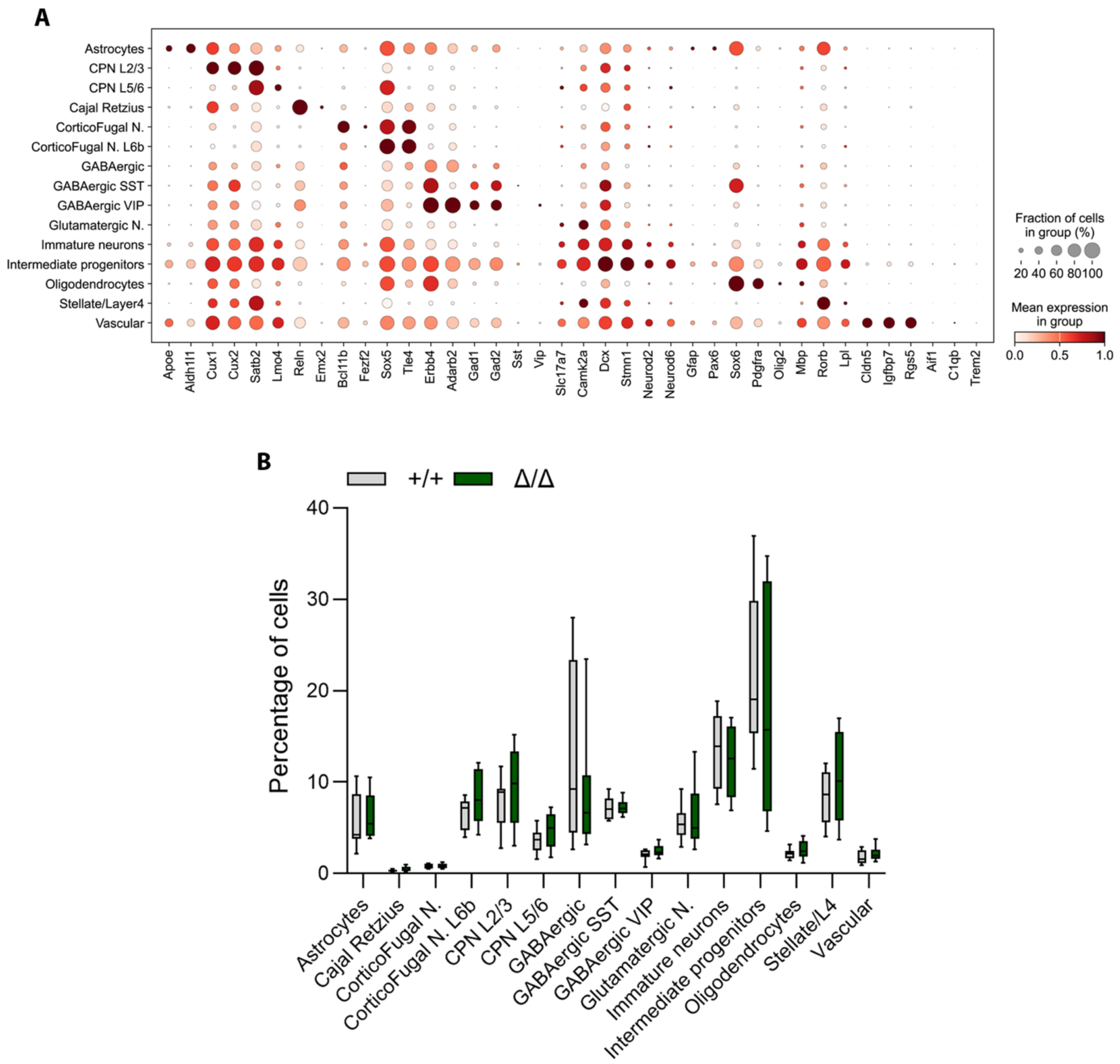


**Fig. S5. Cell markers used for snRNA-seq data analysis.** A) Scaled dot plot depicting the scale expression of key cellular markers used to identify the different cell types in our snRNA-seq experiment B) Analysis of the cellular composition for each genotype does not reveal significant changes in the percentages of each cell population.

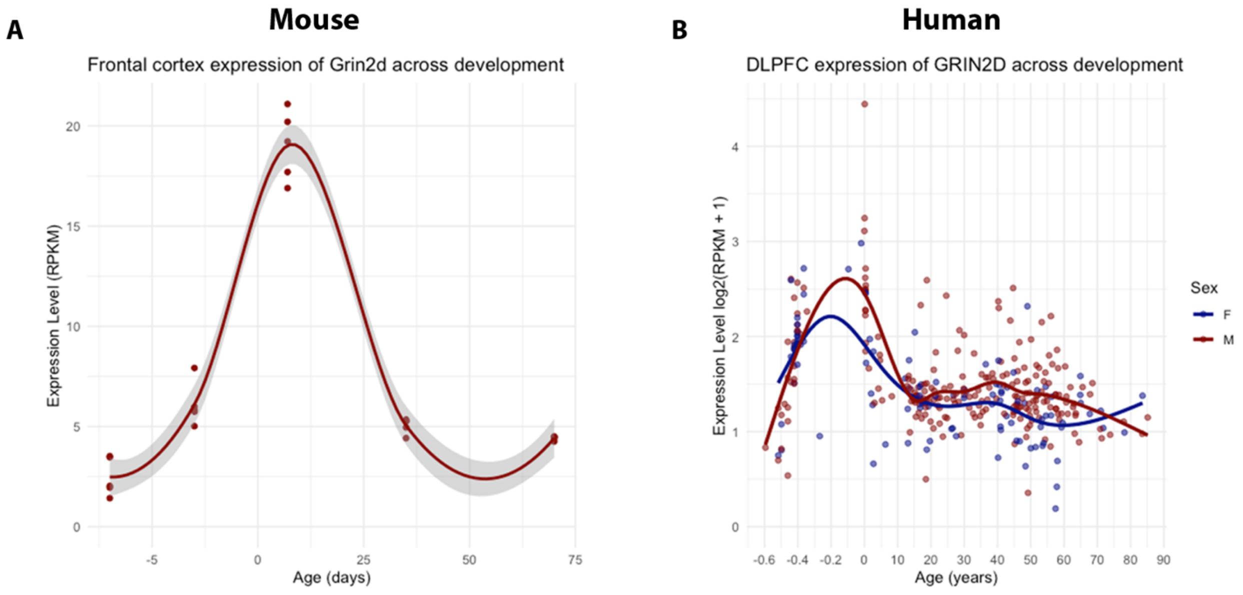


**Fig. S6. *Grin2d* expression is developmentally regulated in the cortex.** **A)** *Grin2d* expression in mouse frontal cortex across development. Data from Clifton et al. (49). **B)** GRIN2D expression in human dorsolateral prefrontal cortex (DLPC) over developmental time. Data from BrainSeq Phase I control post-mortem tissue available at https://eqtl.brainseq.org/phase1/ and based on Jaffe et al Nat Neuro 2018 (48).

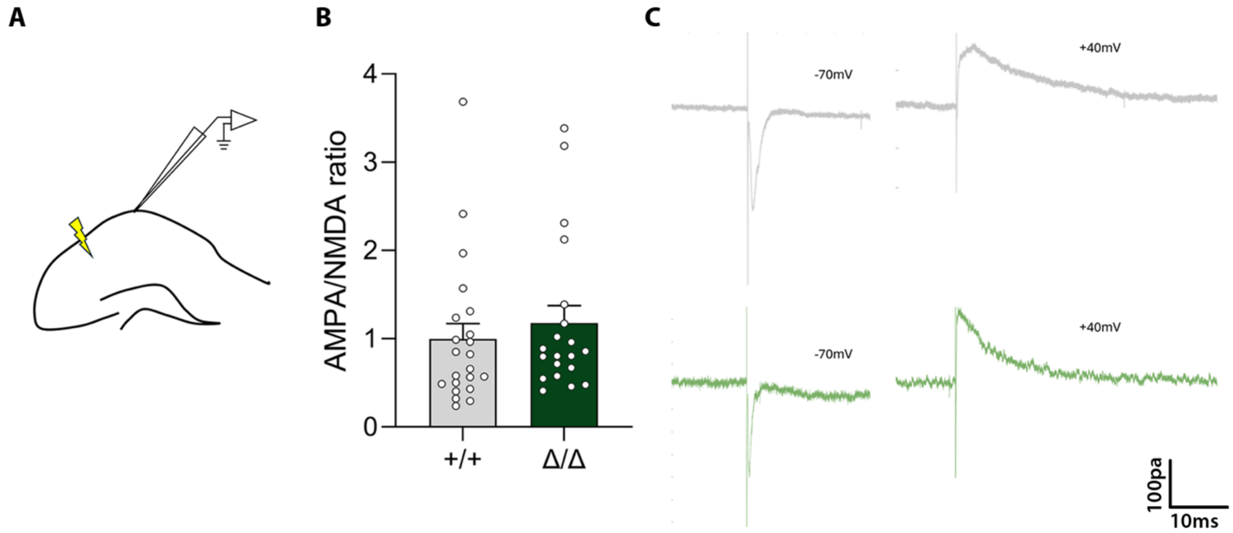


**Fig. S7. *Kdm5b*-mutant mice do not show altered AMPA/NMDA ratios in CA1 pyramidal cells at PND14. A)** Experiment layout with stimulation of the Schaffer collaterals and recoding at CA1. **B)** Bar graph showing the AMPA/NMDA EPSC amplitude ratios (n=22 cells from 8 control mice, and n=20 cells from 4 mutant mice). Data is shown as mean±SEM. **C)** Example traces of AMPA (left) and NMDA (right) responses for both genotypes. Data is represented as mean ± SEM as was analysed with Student's t-test.

**Figure 1F**

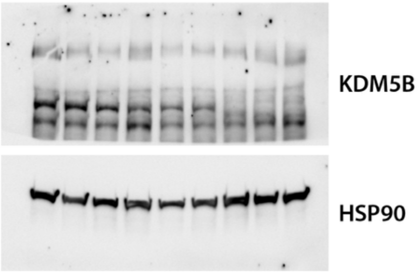


**Figure 1I**

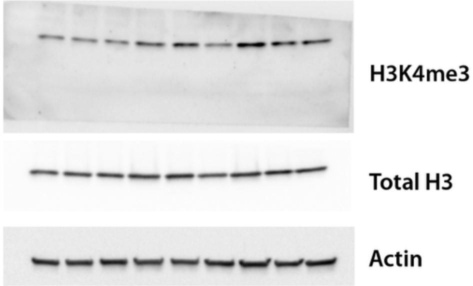


**Figure 7C**

Non-synaptosomal fraction

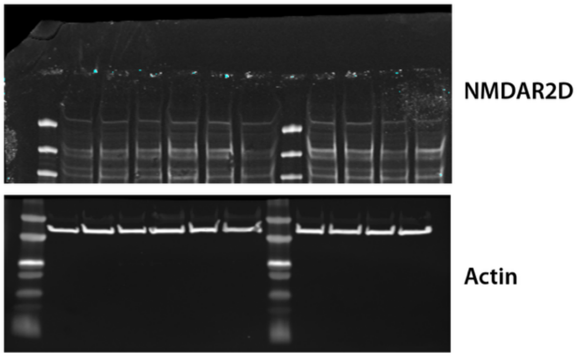


Synaptosomal fraction

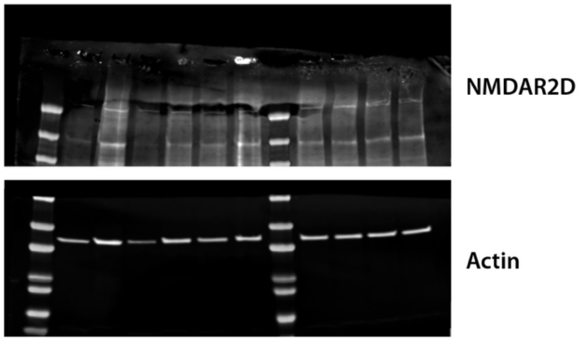


**Fig. S8. Uncropped western blot full scans.** Uncropped full scans of western blots from the corresponding cropped western blot panels shown in Figures 1 and 7.

**Supplementary tables**

**Table S1. Absolute volumes derived from structural MRI analyses in homozygous mutant mice.** MRI analysis revealed significant increases in absolute brain size in different brain regions in homozygous mutant mice.
**Table S2. Relative Absolute volumes derived from structural MRI analyses in homozygous mutant mice.** Correction for absolute brain volume revealed significant changes in volume in different brain regions in homozygous mutant mice.
**Table S3. Absolute volumes derived from structural MRI analyses in heterozygous mutant mice.** Full list of absolute volume of all segmented brain regions.
**Table S4. Relative Absolute volumes derived from structural MRI analyses in heterozygous mutant mice.** Full list of relative volume of all segmented brain regions.
**Table S5. List of differentially expressed genes identified by bulk RNA-sequencing in neocortical samples.** Full list of gene expression changes in PND5 *Kdm5b*$^{\Delta/\Delta}$ neocortex.
**Table S6. List of differentially expressed genes identified by bulk RNA-sequencing in hippocampal samples.** Full list of gene expression changes in PND5 *Kdm5b*$^{\Delta/\Delta}$ hippocampus.
**Table S7. Overlap of differentially expressed genes in the neocortex and hippocampus with the SFARI database.** Full list showing the extent of overlap between DEGs (FDR<0.05), and ASD-associated genes obtained from the SFARI human Gene database.
**Table S8. Overlap of differentially expressed genes in the neocortex and hippocampus with the ID PanelApp database.** Full list showing the extent of overlap between DEGs (FDR<0.05), and ASD-associated genes obtained from the ID PanelApp database.
**Table S9. Primer sequences.** Primers used for quantitative qPCR experiments as shown in Fig. S3.
**Table S10. CUT&Tag diffbind H3K4me3 enrichment (Wild-type vs mutant).** diffBind was used to identify gene TSS with significantly (FDR<0.05) altered enrichment between *Kdm5b*$^{\Delta/\Delta}$ and *Kdm5b*$^{+/+}$ mice.
**Table S11. Overlap of differentially expressed genes identified in the neocortex by bulk RNA-sequencing and those associated with significantly increased enrichment of H3K4me3 proximal to the TSS.** Full list showing the extent of overlap between neocortical DEGs (FDR<0.05), and genes with increased H3K4me3 at TSS identified by CUT&Tag sequencing.
**Table S12. Data values in tabular format.** Source data for figures 1, 2, 3, 7, 8 and supplementary figures 1, 2, 3 and 7.
**Table S13. SFARI genes.** Information retrieved from the SFARI database (accessed October 2025).
**Table S14. ID genes.** Information retrieved from the ID PanelApp database (accessed December 2022).
